# The spread of pyrethroid-resistant mosquitoes and their predicted malaria burden across the Democratic Republic of the Congo

**DOI:** 10.1016/j.crpvbd.2026.100439

**Published:** 2026-09-19

**Authors:** Chadrac Ntembue Tshiakuisha, Andrew C. Glover, Francis Watsenga, Charles S. Wondji, Samuel Bowong, Thomas S. Churcher

**Affiliations:** a Centre for Research in Infectious Diseases, Yaoundé, Cameroon; b University of Douala, Department of Mathematics and Computer Science, Douala, Cameroon; c MRC Centre for Global Infectious Disease Analysis, Imperial College London, London, UK; d Centre Entomologique de Formation, Recherche et Expertise de Kimpese, Kimpese, Democratic Republic of the Congo; e Department of Vector Biology, Liverpool School of Tropical Medicine, Liverpool, UK; f IRD, UMMISCO, Sorbonne University, Bondy, France

**Keywords:** Malaria, Pyrethroid resistance, Insecticide-treated nets, Democratic Republic of the Congo, Mathematical modelling, Vector control

## Abstract

Malaria control in the Democratic Republic of the Congo (DRC) is increasingly challenged by pyrethroid resistance in mosquito populations, although its epidemiological consequences remain uncertain. Entomological data from discriminating-dose bioassays conducted on wild mosquitoes were collated to characterise temporal changes in pyrethroid resistance since 2009. These estimates were incorporated into a mechanistic malaria transmission model to quantify how resistance may have reduced the effectiveness of insecticide-treated nets (ITNs) and affected malaria burden. Model projections were compared with malaria prevalence estimates from the 2013–2014 and 2023–2024 Demographic and Health Surveys, which were used as external comparisons rather than for model fitting. Counterfactual scenarios were generated in which mosquitoes remained fully susceptible to pyrethroids throughout the study period. The model estimated that pyrethroid resistance increased substantially over time and reduced the protection provided by pyrethroid-only ITNs. Nationally, approximately 8.6% of malaria clinical cases, corresponding to 33.6 million additional cases, and approximately 76,000 additional deaths between 2009 and 2024 were attributable to pyrethroid resistance in the modelled counterfactual comparison. The introduction of pyrethroid-PBO ITNs from 2019 partially restored the protection lost to pyrethroid resistance. These findings highlight the potential epidemiological consequences of pyrethroid resistance while also emphasising the uncertainty arising from sparse resistance surveillance and limited epidemiological observations. Improved entomological surveillance and continued deployment of more effective vector-control tools will be important for malaria control in the DRC.

## Highlights

• Pyrethroid resistance increased substantially over time in the Democratic Republic of the Congo.
• Resistance substantially reduced the protection provided by pyrethroid-only ITNs.
• Pyrethroid-PBO ITNs partially restored protection lost to pyrethroid resistance.
• Modelling links resistance trends to national malaria cases and deaths.

## Graphical abstract

**Figure:**
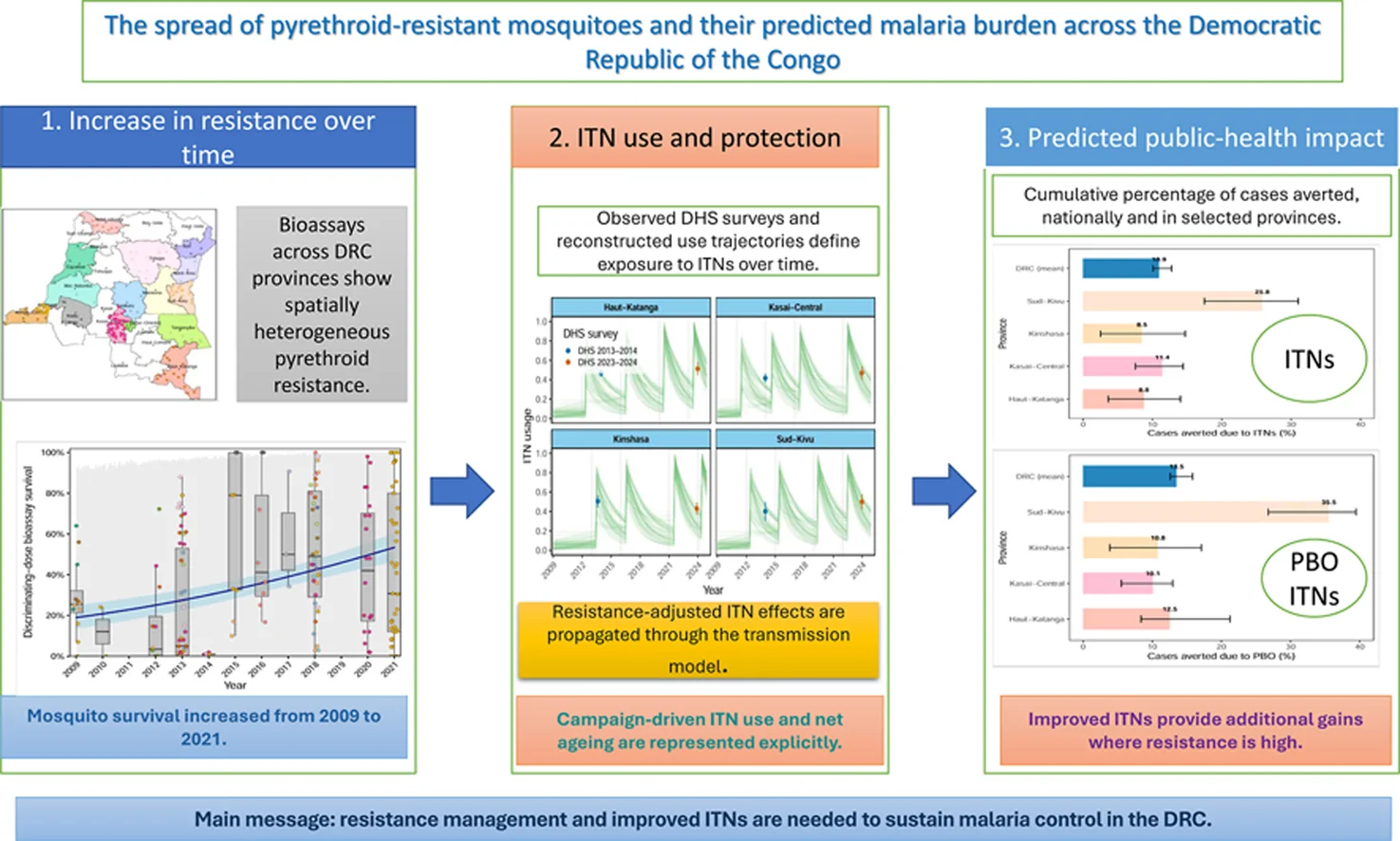

## 1 Introduction

In tropical and subtropical regions of the world, malaria continues to be one of the most serious infectious diseases (WHO, 2019, 2025). The disease is still a major public health concern, particularly in sub-Saharan Africa, despite notable improvements over the last 20 years (WHO, 2025). According to recent estimates, this region has the highest number of malaria deaths and cases worldwide (WHO, 2025). The Democratic Republic of the Congo is consistently among the largest contributors to the global malaria burden (WHO, 2025). According to the World Health Organisation, there were an estimated 282 million malaria cases and 610,000 malaria-related deaths globally in 2024. Approximately 95% of these cases and fatalities occurred in the African region (WHO, 2025). An estimated 35 million cases and 68,000 fatalities were attributed to the DRC in the same year, accounting for 12.5% of all recorded malaria cases and 11.1% of malaria-related deaths worldwide (WHO, 2025). These numbers highlight the substantial malaria burden in the Democratic Republic of the Congo, where the disease continues to be a major cause of morbidity and mortality. Furthermore, the country remains an important focus for international efforts to control and eliminate malaria because of its epidemiological importance.

In the Democratic Republic of the Congo (DRC), insecticide-treated nets (ITNs) remain one of the few malaria control tools that can be delivered at scale and used consistently in rural and urban settings alike (Wat’senga et al., 2018; WHO, 2025). The large-scale rollout of ITNs over the past 20 years, particularly pyrethroid-treated nets, has been credited with many of the gains seen in malaria reduction across sub-Saharan Africa (Bhatt et al., 2015). Yet, the same success has brought a new challenge. Evidence indicates that resistance to pyrethroids, the insecticide class historically used in conventional ITNs, is now widespread across many parts of the DRC and threatens to undermine the progress achieved in malaria control (Wat’senga et al., 2018, 2020).

Resistance arises through different mechanisms. Some mechanisms cause the insecticide to break down before it can act, while others make the target sites less sensitive (WHO, 2025). It has been suggested that resistance in mosquitoes appears first in areas with the highest ITN coverage (Hancock et al., 2020). Studies from the DRC have documented widespread pyrethroid resistance and have raised concerns about the reduced effectiveness of pyrethroid-only ITNs in some settings (Programme National de Lutte contre le Paludisme, 2012; WHO, 2025). However, geographical differences in malaria burden cannot be attributed to insecticide resistance alone. Variation in transmission intensity, ITN access and use, mosquito behaviour, environmental conditions, and the timing and quality of intervention delivery may also influence the observed burden. Evidence from the DRC and other malaria-endemic countries nevertheless indicates that increasing pyrethroid resistance can reduce the entomological and epidemiological effectiveness of pyrethroid-only ITNs (Programme National de Lutte contre le Paludisme, 2012; Nash et al., 2021; WHO, 2021). To respond to this growing resistance, the WHO no longer recommends relying on a single type of ITN. Instead, three newer net types are now recommended. The strongest recommendation is for nets that combine a pyrethroid with the pyrrole insecticide chlorfenapyr. Nets treated with a pyrethroid plus the synergist piperonyl butoxide (PBO) are also supported, particularly where metabolic resistance is common. A third option combines a pyrethroid with the growth regulator pyriproxyfen (PPF), which offers some additional benefit over pyrethroid-only ITNs but is not expected to replace other nets (WHO, 2025). The additional benefit of these different nets in the DRC context is currently unknown. Malaria cases are reported each year through the national health information system, although these records do not capture every case. Cases may be missed when people do not attend a reporting health facility or are not tested, and the completeness of reporting varies between locations. The WHO estimates of the national malaria burden therefore combine routine surveillance records with household survey data and statistical modelling to account for cases that are not recorded by the health system (WHO, 2025). Only two national surveys have been conducted in the DRC since 2007, in 2013–2014 and 2023–2024, making it hard to understand the epidemiological impact of ITNs or the consequences of mosquito pyrethroid resistance.

Discriminating-dose bioassays provide a simple method for assessing the susceptibility of the local mosquito population to different insecticides and have become the main source of resistance surveillance data across Africa (Ranson and Lissenden, 2016; WHO, 2016). Mosquitoes are exposed to a defined concentration of insecticide and the proportion surviving provides a measure of resistance. The insecticide concentration used in a bioassay is intended to be lower than the exposure experienced by mosquitoes during attempted blood-feeding in a natural setting to identify resistance before it starts to have a public health impact. The current best method for assessing the entomological impact of insecticide resistance is to test ITNs in experimental hut trials, which allow wild, free-flying mosquitoes to interact with ITNs. A nonlinear model developed by Nash et al. (2021) links mosquito survival measured in discriminating-dose bioassays with survival following exposure to an ITN in experimental hut trials (Sherrard-Smith et al., 2022b). This relationship provides a way to translate laboratory measurements of pyrethroid resistance into expected changes in mosquito survival under more field-relevant conditions. Mathematical models of malaria transmission dynamics parameterised with the results of discriminating-dose bioassays were able to reproduce the differences in malaria prevalence observed between standard pyrethroid ITNs and pyrethroid-PBO nets in cluster randomised controlled trials (RCTs) (Sherrard-Smith et al., 2022b).

Mathematical models provide a tool for exploring the expected impact of different malaria interventions across a range of programmatic outcomes (Griffin et al., 2010) and offer a practical way to estimate how insecticide resistance may alter intervention effectiveness without conducting new, expensive trials in every setting (Churcher et al., 2016; Nash et al., 2021). Mathematical models also provide a framework for exploring counterfactual scenarios and assessing how changes in ITN use and pyrethroid resistance affect malaria burden at the population level. In this study, we extend these approaches to the DRC by combining statistical analysis of discriminating dose data with transmission dynamics mathematical models to investigate how mosquito resistance may have impacted malaria epidemiology between 2009 and 2024.

## 2 Materials and methods

### 2.1 Quantifying changing resistance over time

Insecticide-resistance observations for the DRC were obtained from the WHO Malaria Threats Map for 2009–2020 (WHO, 2020). Additional observations for 2021 were obtained from Nguiffo-Nguete et al. (2023). The outcome extracted from each discriminating-dose bioassay was the proportion of mosquitoes surviving exposure to the insecticide. The number of mosquitoes tested was not reported for 29 of the 205 bioassays, corresponding to 14.1% of the dataset. Among the remaining 176 bioassays for which the sample size was available, 109 (61.9%) tested exactly 100 mosquitoes, making this the most commonly reported sample size. In the primary analysis, the 29 observations with missing sample-size information were therefore assigned a value of 100 mosquitoes, corresponding to four replicates of 25 mosquitoes under the standard WHO bioassay protocol. The robustness of the estimated resistance trajectory to this assumption was assessed by repeating the analysis using alternative sample sizes of 50 and 83 mosquitoes, as described in Supplementary Text S1 (Section A.1) and shown in Supplementary Fig. S2. Information on the species of mosquitoes examined is generally lacking from the WHO dataset. In the absence of consistent species information, observations from all tested *Anopheles* spp. mosquitoes were pooled to estimate temporal changes in bioassay survival. In total, the dataset consisted of 16,934 mosquitoes analysed in 205 bioassays. The distribution of observations across the study period is summarised in Supplementary Table S2.

### 2.2 Statistical model of pyrethroid-resistance trends

The discriminating-dose bioassay data demonstrated considerable variability in observed percentage mortality, even within relatively small temporal and geographical areas. The data were therefore analysed using a beta-binomial likelihood. Let $X_{i}$ denote the number of mosquitoes surviving bioassay i, $N_{i}$ the number tested, and $t_{i}$ the year in which the bioassay was conducted. We assumed

$$X_{i}∼\text{Beta}-\text{Binomial}\left(N_{i}|\alpha_{i},\beta_{i}\right)\text{,}$$

where $\alpha_{i}=m\left(t_{i}\right)k$, $\beta_{i}=\left[1-m\left(t_{i}\right)\right]k$, $m\left(t_{i}\right)$ is the estimated mean probability that a mosquito survives the discriminating-dose bioassay at time $t_{i}$, and k is the beta-binomial precision parameter. Larger values of k indicate that the variation between observations approaches that expected under a binomial distribution, whereas smaller values indicate greater variation around the estimated mean survival probability. Temporal change in mean bioassay survival was described using a three-parameter generalised logistic function,

$$m\left(t\right)={\left[1+\exp\left\{-a\left(t-b\right)\right\}\right]}^{-1/c}\text{,}$$

where t was measured in years, a governs the rate of change in survival, b determines the location of the inflection point, and c controls the asymmetry of the trajectory. This functional form was specified a priori and was not selected through a formal comparison of alternative temporal functions.

Posterior inference was performed in Stan using Hamiltonian Monte Carlo (HMC) (Carpenter et al., 2017). Eight chains were run for 6000 iterations each, with a 2000-iteration warm-up. Convergence was assessed using the Gelman-Rubin diagnostic, with all parameters exhibiting $\hat{R}<1.1$ (Vats and Knudson, 2021). Trace plots and posterior summaries were inspected following standard Stan diagnostic guidance (Carpenter et al., 2017).

Differences in the number of mosquitoes tested were included directly in the likelihood. Laboratory, replicate and seasonal effects were not modelled explicitly because these characteristics were unavailable or were not reported consistently across the source datasets. The beta-binomial likelihood accounted for unexplained variation between bioassays, but the available data did not allow the separate sources of this variation to be identified. Sensitivity analyses were conducted to examine whether the national trajectory was disproportionately influenced by observations from Kinshasa or other well-sampled provinces. These analyses are described in Supplementary Text S1 (Section A.2) and shown in Supplementary Fig. S1. Parameter priors and posterior estimates for the national resistance model are summarised in Supplementary Table S3.

### 2.3 Translating bioassay survival into ITN entomological effects

Discriminating-dose bioassay survival was translated into the initial deterrent, mosquito-killing and blood-feeding effects of pyrethroid-only and pyrethroid-PBO ITNs using previously published relationships between bioassay and experimental hut trial outcomes (Nash et al., 2021; Churcher et al., 2024). No additional model was fitted to the DRC bioassay observations at this stage. Resistance was evaluated when each new ITN cohort was distributed and determined the initial efficacy assigned to that cohort. Net efficacy subsequently declined with age but was not updated in response to later changes in resistance. Nets distributed in different years could therefore have different initial efficacies, reflecting the estimated resistance level at the time of deployment. Uncertainty in the resistance trajectory and the bioassay-to-hut relationships was propagated through the transmission simulations. Parameter definitions and sources are provided in Supplementary Text S1 (Section A.4) and Supplementary Table S3. Initial entomological efficacy parameters are summarised in Supplementary Table S1, and the bioassay-to-hut relationship is shown in Supplementary Fig. S3.

### 2.4 Mathematical model of malaria transmission

We used a malaria transmission framework originally developed by Griffin et al. (2010) and subsequently extended to represent the effects of pyrethroid resistance on different ITNs (Churcher et al., 2016; Sherrard-Smith et al., 2022a). The model was implemented using the R packages *malariasimulation* (v.3.0.0) and *malariaEquilibrium*, which are publicly accessible at https://mrc-ide.github.io/malariasimulation/. Further details of the model structure and parameterisation are available in the original model publications (Griffin et al., 2010; White et al., 2011) and the Supplementary Text S1 (Section A.8). Annual population denominators were obtained from the *foresite* package (Winskill, 2024). Administrative boundaries were harmonised to the current 26-province structure, and the analysis was restricted to 2009–2024 because ITN use before this period was thought to be minimal. Baseline transmission intensity and demographic and intervention inputs were specified separately for each province. Provincial entomological inoculation rates were selected to reproduce malaria prevalence before the modelled intervention history, using prevalence estimates and associated covariates obtained through the *foresite* package from Malaria Atlas Project estimates. Because resistance observations were too sparse and unevenly distributed to estimate reliable provincial trajectories, the same sampled national resistance trajectory was applied to all provinces. Provincial prevalence was aggregated to the national level using population-weighted means, whereas provincial cases and deaths were aggregated by summation. One hundred posterior simulations were conducted for each province and intervention scenario. Further details of the provincial inputs and propagation of uncertainty are provided in the Supplementary Text S1 (Supporting Methods).

### 2.5 Modelled intervention scenarios

The reference scenario represented the estimated history of ITN deployment, including the mixture of pyrethroid-only and pyrethroid-PBO ITNs and the nationally estimated temporal change in pyrethroid resistance. Three counterfactual scenarios were considered. In the no-resistance scenario, the same ITN-use trajectory, campaign timing, population, transmission intensity and other model inputs were retained, but mosquitoes were assumed to remain fully susceptible to pyrethroids throughout the study period. Initial ITN efficacy parameters were therefore fixed at values expected for a fully susceptible mosquito population. In the no-ITN scenario, ITN use was set to zero while all other non-ITN inputs were retained. This scenario was used to estimate the overall impact of ITNs. In the pyrethroid-only counterfactual, ITN use and campaign timing were retained, but all nets distributed from 2019 onwards were assumed to be pyrethroid-only ITNs. Comparison with the reference scenario was used to estimate the additional impact of introducing pyrethroid-PBO ITNs.

For each transmission-model simulation, a complete resistance trajectory was sampled from the posterior distribution of the national bioassay model, preserving the temporal correlation between successive annual estimates. Because the final bioassay observations were collected in 2021, resistance values for 2022–2024 were obtained from the corresponding sampled posterior trajectory. Uncertainty associated with this extrapolation was retained in the transmission simulations.

### 2.6 Calibration of malaria cases and deaths

The scenario representing observed ITN deployment and the estimated level of pyrethroid resistance was used as the reference scenario. Let $C_{r,s,t,d}^{\text{raw}}$ denote the uncalibrated number of malaria cases predicted for province r, scenario s, year t, and simulation draw d. The corresponding national total was obtained by summing across provinces:

(3) $$C_{s,t,d}^{\text{raw},\text{nat}}=\sum_{r}C_{r,s,t,d}^{\text{raw}}\text{.}$$

For the reference scenario,

(4) $$C_{\text{ref},t,d}^{\text{raw},\text{nat}}=\sum_{r}C_{r,\text{ref},t,d}^{\text{raw}}\text{.}$$

To align model-derived case numbers with WHO national malaria-burden estimates, a year- and draw-specific calibration factor was calculated as

(5) $$\alpha_{t,d}=\frac{C_{t}^{\text{WHO}}}{C_{\text{ref},t,d}^{\text{raw},\text{nat}}}\text{,}$$

where $C_{t}^{\text{WHO}}$ is the WHO national estimate of malaria cases in year t. The same annual calibration factor was applied to the reference and counterfactual scenarios

(6) $${\;C}_{r,s,t,d}^{\text{cal}}=\alpha_{t,d}C_{r,s,t,d}^{\text{raw}}\text{.}$$

Consequently,

(7) $$\sum_{r}C_{r,\text{ref},t,d}^{\text{cal}}=C_{t}^{\text{WHO}}\text{.}$$

The reference scenario was therefore constrained to reproduce the WHO national case estimate in each year and simulation draw, while relative differences between scenarios were determined by the transmission model. Malaria prevalence was not calibrated to WHO case estimates and was retained as a direct transmission-model output. Deaths were estimated using the annual WHO case-fatality ratio,

(8) $${\text{CFR}}_{t}=\frac{D_{t}^{\text{WHO}}}{C_{t}^{\text{WHO}}}\text{,}$$

where $D_{t}^{\text{WHO}}$ denotes the WHO national estimate of malaria deaths in year t. Calibrated deaths were then calculated as

(9) $$D_{r,s,t,d}^{\text{cal}}={\text{CFR}}_{t}C_{r,s,t,d}^{\text{cal}}\text{.}$$

The annual calibration factors, WHO case-fatality ratios, and calibrated national case and death estimates are presented in Supplementary Table S4.

### 2.7 Model checking

Posterior predictive checks were used to assess whether the resistance model reproduced the observed variation between bioassays. Reconstructed ITN-use trajectories and modelled malaria prevalence were also compared with available DHS observations. The DHS prevalence estimates were not used to fit the transmission model and were treated as an external comparison rather than as formal model validation. Further details are provided in Supplementary Text S1 (Sections A.3 and A.10). The numbers tested and positive by rapid diagnostic test in the 2013–2014 and 2023–2024 DHS surveys are reported in Supplementary Tables S5 and S6, respectively.

## 3 Results

### 3.1 Characterising resistance trends

The availability of bioassay data varied over time and across the country, with some provinces contributing few or no observations (Fig. 1A). Despite this uneven distribution of data, the estimated level of pyrethroid resistance in the mosquito population increased across the DRC between 2009 and 2021 (Fig. 1B). Model-based estimates show that the level of resistance, as defined as the probability that a mosquito survives 24 h after exposure to an insecticide in a discriminating-dose bioassay, steadily increased from an average of 17.4% survival in 2009, 33.3% in 2015, 44.8% in 2018, and finally to 57.2% in 2021. By the end of the study, the national average resistance levels were approximately 3.3 times the estimated 2009 level. There is considerable heterogeneity within each year, with bioassay results varying from 0 to 100% mortality within the same province in the same year. The type of ITN distributed also varied, with pyrethroid-PBO ITNs being introduced in 2019 and becoming the dominant ITN in use by 2024 (Fig. 1C).

**Fig. 1:**
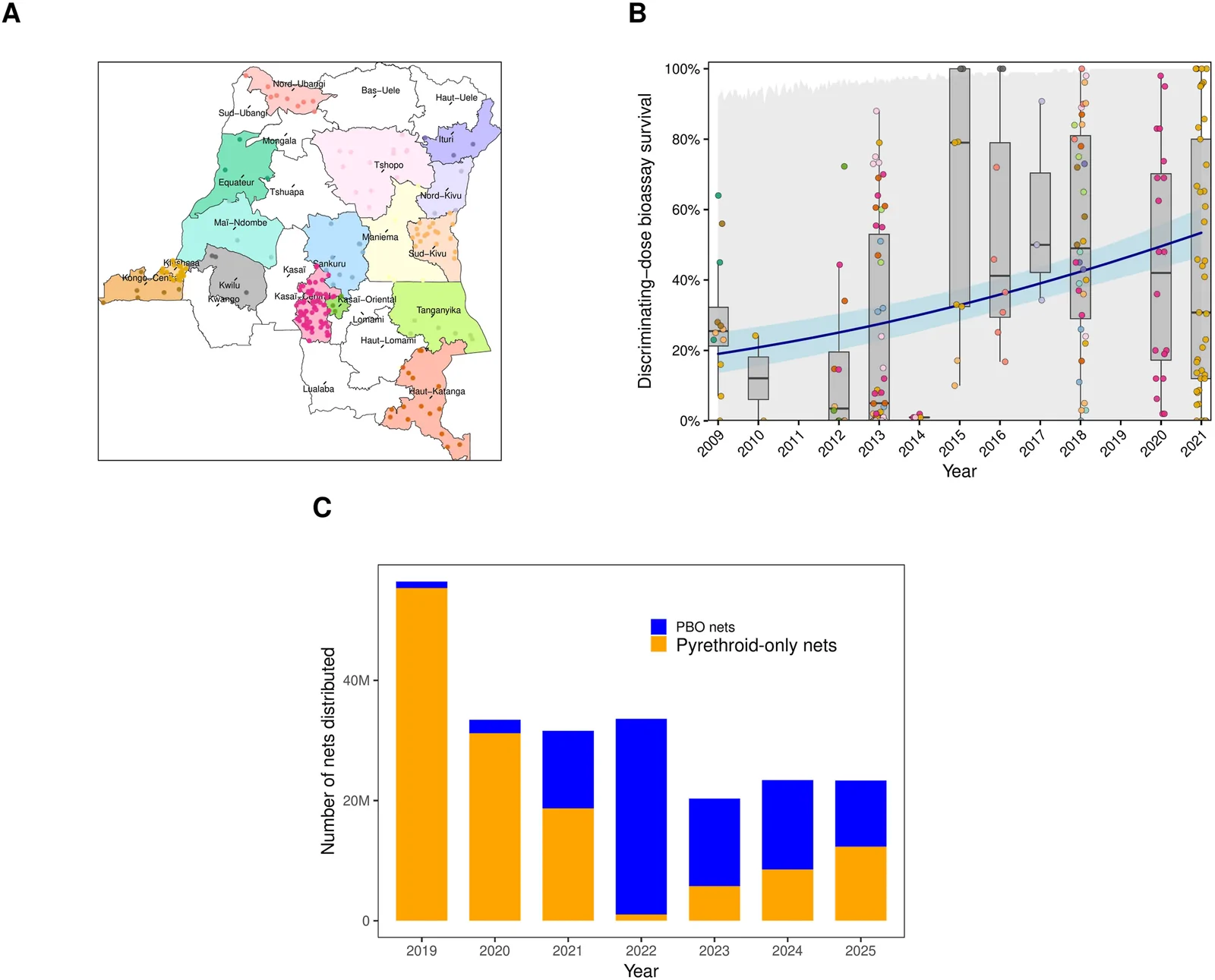
The level of pyrethroid resistance in mosquitoes in the Democratic Republic of the Congo (DRC). **A** Map illustrating the sites where insecticide resistance data were sampled across the country. Each dot represents the location where mosquitoes were sampled for a single bioassay, with colours denoting the province of sampling. **B** The temporal change in pyrethroid resistance. Coloured points represent the observed proportion of mosquitoes surviving each discriminating-dose bioassay and correspond to the provinces shown in panel **A**. Boxplots summarise the median, interquartile range and distribution of the observed survival values within each year. The solid blue line represents the posterior median fitted national resistance trajectory, and the blue shaded region shows the corresponding 95% credible interval for the underlying mean trajectory. The grey shaded region shows the 95% posterior predictive interval for individual bioassay observations generated from the beta-binomial model. **C** Comparison of the yearly distribution of PBO ITNs with pyrethroid-only ITNs in the DRC.

### 3.2 Overall ITN use

From 2009 to 2024 NMCP reports indicated that provinces such as Bas-Uélé, Kasaï-Oriental, Kwango, Kwilu, Lomami, Tshopo, Sud-Kivu, Nord-Kivu, Équateur, Haut-Katanga, Ituri, Kinshasa, and Maniema implemented four mass ITN distribution campaigns, whereas Haut-Lomami, Lualaba, Nord-Ubangi, Sud-Ubangi, and Tanganyika recorded three. In several areas, inter-campaign intervals exceeded six years, and in parts of Ituri and Mongala, gaps approached eight years. Month-specific campaign timings were identified where records were available, typically occurring between June and September. Details for the remaining provinces are provided in Supplementary Fig. S4–S6. Additional summaries of provincial ITN use and the proportion of ITNs distributed through routine channels are provided in Supplementary Figs. S16 and S17, respectively.

DHS data collected from 2013–2014 and 2023–2024 are broadly consistent with the Africa-wide estimated growth in ITN use and mean net retention time of 19.8 months (95% CrI: 13.5–34.9) reported in Glover et al. (2026). There is some variability between regions not captured in the model (Fig. 2A) but DHS point estimates are generally within the uncertainty ranges explored. Across the Democratic Republic of the Congo at a national level, the model predicts an average ITN use of 48.6% (95% CrI: 33.0–62.2%) between 2023 and 2024 which is in line with the DHS estimates of 47.0% collected in the same period.

**Fig. 2:**
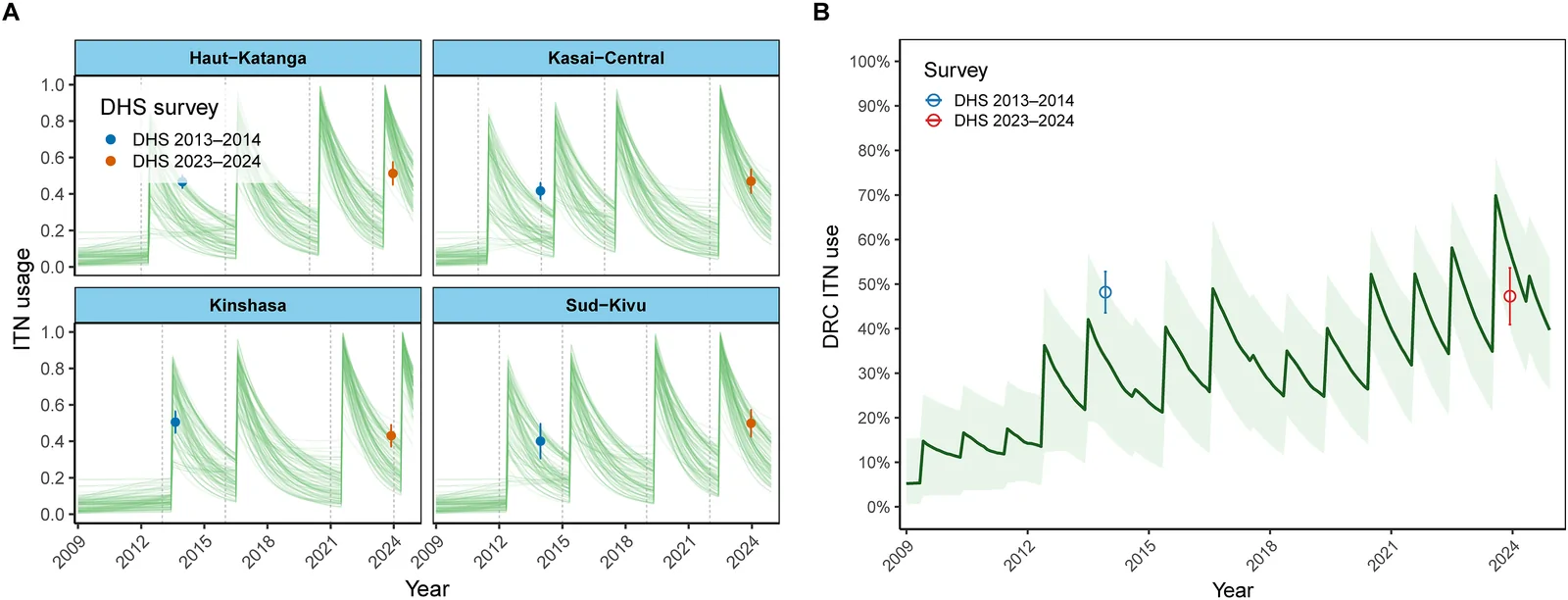
Subnational time series of predicted ITN use in the DRC between 2009 and 2024. Panel A shows estimated ITN use over time in selected provinces of the DRC. The green lines depict 100 posterior trajectories from the Africa-wide model estimated in Glover et al. (2026), illustrating uncertainty in the model. Coloured points show province-specific DHS estimates from the 2013–2014 survey (*blue*) and the 2023–2024 survey (*red*), with vertical bars indicating the reported uncertainty. Vertical dashed lines denote the timing of mass ITN distribution campaigns based on National Programme reports. Corresponding plots for all provinces in the DRC are provided in Supplementary Figs. S4–S6. **B** The national ITN use over time, with the green line representing the average across all 26 provinces.

### 3.3 Predicted malaria prevalence with or without resistance

The model was used to predict malaria prevalence in the DRC under the resistance scenario and under a counterfactual scenario in which mosquitoes remained fully susceptible to pyrethroids throughout the study period (Fig. 3A and B). Under observed resistance patterns, the model predicts that ITNs substantially reduce malaria prevalence in the years after each mass ITN distribution, with their impact increasing as ITN use increases over time. Four provinces together with the national average are shown for clarity, and equivalent trajectories for all 26 provinces are provided in Supplementary Figs. S7–S9. Under observed resistance, the estimated prevalence exceeded 40% in several provinces, including Kasaï-Central, Kasaï, Kwango, Lomami, Mongala, Nord-Ubangi, Sud-Ubangi, Tanganyika, Tshuapa, Bas-Uélé, Haut-Uélé, and Ituri, while the predicted prevalence in Kinshasa, Haut-Katanga, Kasaï-Oriental, and surrounding provinces was substantially lower. Between 2019 and 2024, only a single mass distribution of pyrethroid-PBO nets was recorded in several provinces, including Kasaï, Kwilu, Kwango, Maniema, Mai-Ndombe, Nord-Ubangi, Tanganyika and Kasaï-Central. The model reproduced the malaria prevalence estimates from the two DHS surveys relatively poorly (Fig. 3A and B).

**Fig. 3:**
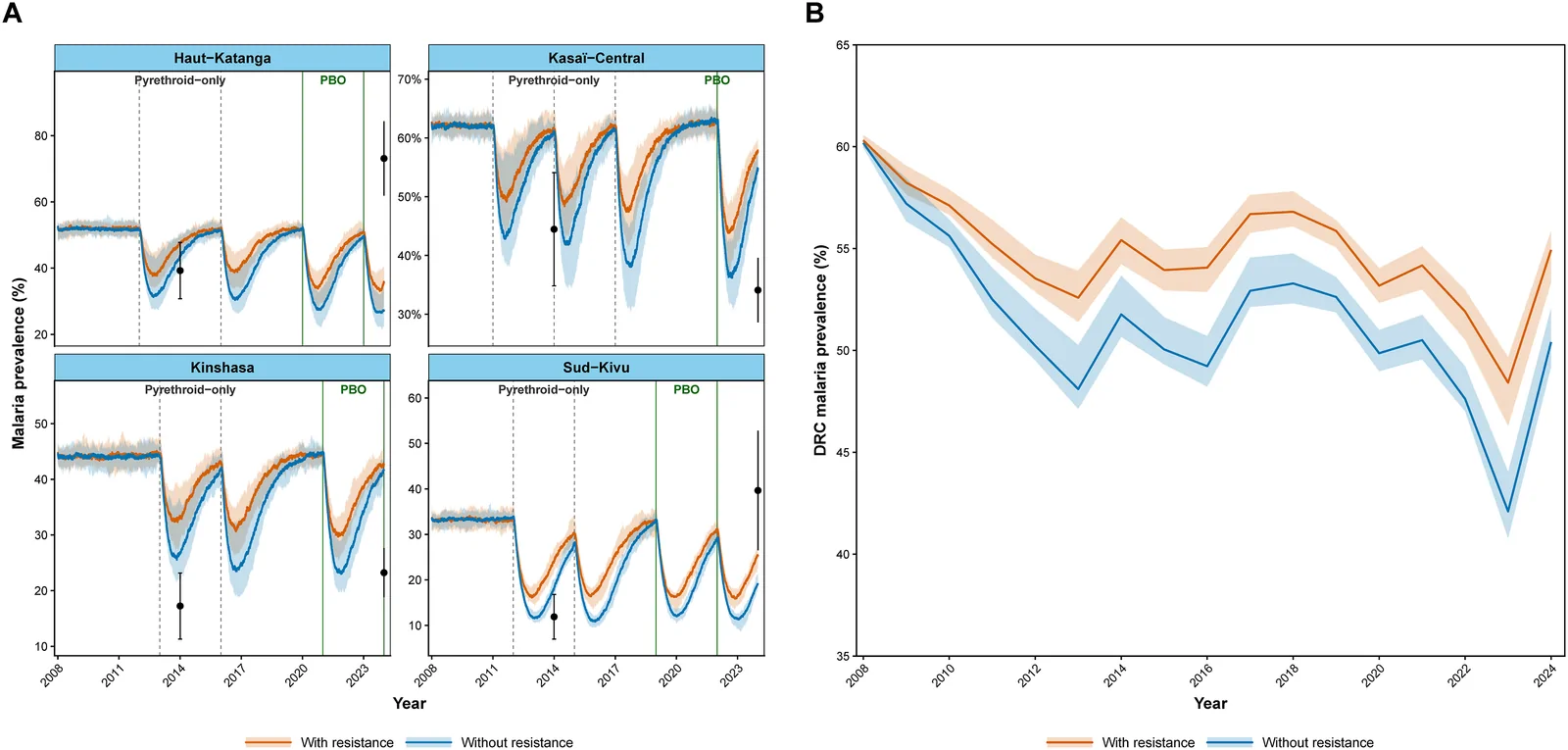
Temporal trends in malaria prevalence under scenarios with and without pyrethroid resistance in the mosquito population. **A** Projected malaria prevalence in children under 5 years of age, measured by microscopy, in four provinces of the Democratic Republic of the Congo (DRC). The orange line represents the reference scenario incorporating the estimated temporal change in pyrethroid resistance, while the blue line represents the counterfactual scenario in which mosquitoes remained fully susceptible to pyrethroids throughout the study period. Points show province-specific prevalence estimates from the 2013–2014 and 2023–2024 Demographic and Health Surveys (DHS). Vertical dashed lines indicate mass distribution campaigns using pyrethroid-only insecticide-treated nets (ITNs), while solid green vertical lines indicate campaigns that introduced pyrethroid-PBO ITNs. Shaded bands represent the 95% credible intervals obtained from the posterior transmission-model simulations. Results for the remaining provinces are presented in Supplementary Figs. S7–S9. **B** Population-weighted national mean malaria prevalence in the DRC under the reference and no-resistance counterfactual scenarios. Shaded bands represent the corresponding 95% credible intervals.

These survey estimates were not used to fit the transmission model and are presented as an external comparison. The prevalence projections should therefore be interpreted primarily in terms of the relative difference between the resistance and no-resistance scenarios, rather than as precise predictions of national prevalence in the survey years. The discrepancy between the model projections and the survey estimates may partly reflect uncertainty in the reconstructed ITN-use and insecticide-resistance trajectories, neither of which fitted the available data particularly well. The national model may also obscure geographical variation, as malaria prevalence increased between the two surveys in some provinces, such as Haut-Katanga, but declined in others (Fig. 3A). At the national level, the models predicted that ITNs, after accounting for the estimated level of pyrethroid resistance, reduced malaria prevalence by an average of 9.9% over the study period. In 2024, malaria prevalence was predicted to be 12.4% lower than it would have been if no ITNs had been distributed, all else being equal (Fig. 3B).

As expected, the counterfactual scenario which assumes that mosquitoes did not develop pyrethroid resistance predicts ITNs would have been more impactful and resulted in a lower malaria prevalence. Nevertheless, the model predicted a relatively modest difference in malaria prevalence between the resistance and no-resistance scenarios. Across 2009–2024, national malaria prevalence was approximately 4 percentage points higher, on average, when resistance was included, corresponding to a mean relative increase of approximately 7%. The difference varied over time and was greatest in 2023, when the absolute difference reached approximately 7 percentage points (Fig. 3B). Impact varied substantially; Sud-Kivu exhibited the largest relative rise, with prevalence roughly 25–28% higher than the counterfactual. Kinshasa showed a moderate increase of about 10%, Kasaï-Central about 7–8%, and Haut-Katanga the lowest at 5%.

In the model projections, the difference between the resistance and no-resistance scenarios was generally smallest immediately after ITN campaigns and increased later in each campaign cycle, although the magnitude and timing of this pattern remain uncertain. Within the model simulations, pyrethroid-PBO ITNs reduced the effect of resistance, although predicted prevalence remained higher than in the fully susceptible counterfactual scenario. The estimates therefore reflect relative differences between scenarios rather than precise predictions of national malaria prevalence.

### 3.4 Cases and deaths averted by ITN use

The model predicted the proportion of cases averted by ITNs (compared to a no ITN distribution) ranged from approximately 8.5% to 25.8% across the four illustrated provinces (Fig. 4A) and between 3.8% and 20.8% for the other provinces. The national mean was around 10.9%. Sud-Kivu showed the largest reduction (around 25.8%), followed by Kasaï-Central (around 11.4%), while Kinshasa and Haut-Katanga had lower estimates (around 8.5% and 8.8%, respectively). Uncertainty was widest for Kinshasa and Haut-Katanga relative to the other series. The corresponding estimates for the remaining 22 provinces are reported in Supplementary Fig. S10. Nationally, the modelled percentage of cases averted increased over time, from around 1% (2011) to approximately 13–14% (2015), remained broadly within 7–12% through 2016–2021, rose to around 16–17% (2022), peaked at approximately 22–23% (2023), and was around 12–13% (2024) (Fig. 4E). This suggests that the increased usage over time offsets any loss of efficacy over this period due to the increase in pyrethroid resistance. Estimates of deaths averted by ITN use increased from < 0.1 million (2011) to approximately 1.0 million (2015), fluctuated between roughly 0.6 and 1.0 million during 2016–2021, rose to around 1.4 million (2022), peaked near 1.9 million (2023), and were approximately 1.0–1.1 million (2024) (Fig. 4F). Values fluctuate year on year as the greatest number of cases averted occurs the year preceding the mass campaign, with 2023 seeing mass campaigns in Kasaï, Haut-Lomami, Ituri, Kasaï-Oriental, Lualaba, Mai-Ndombe, Tshopo, Sud-Ubangi, Mongala, Nord-Ubangi and Tanganyika provinces compared to Bas-Uélé and Kinshasa provinces in 2024. Province-level values contributing to these national totals are reported in Supplementary Fig. S12.

**Fig. 4:**
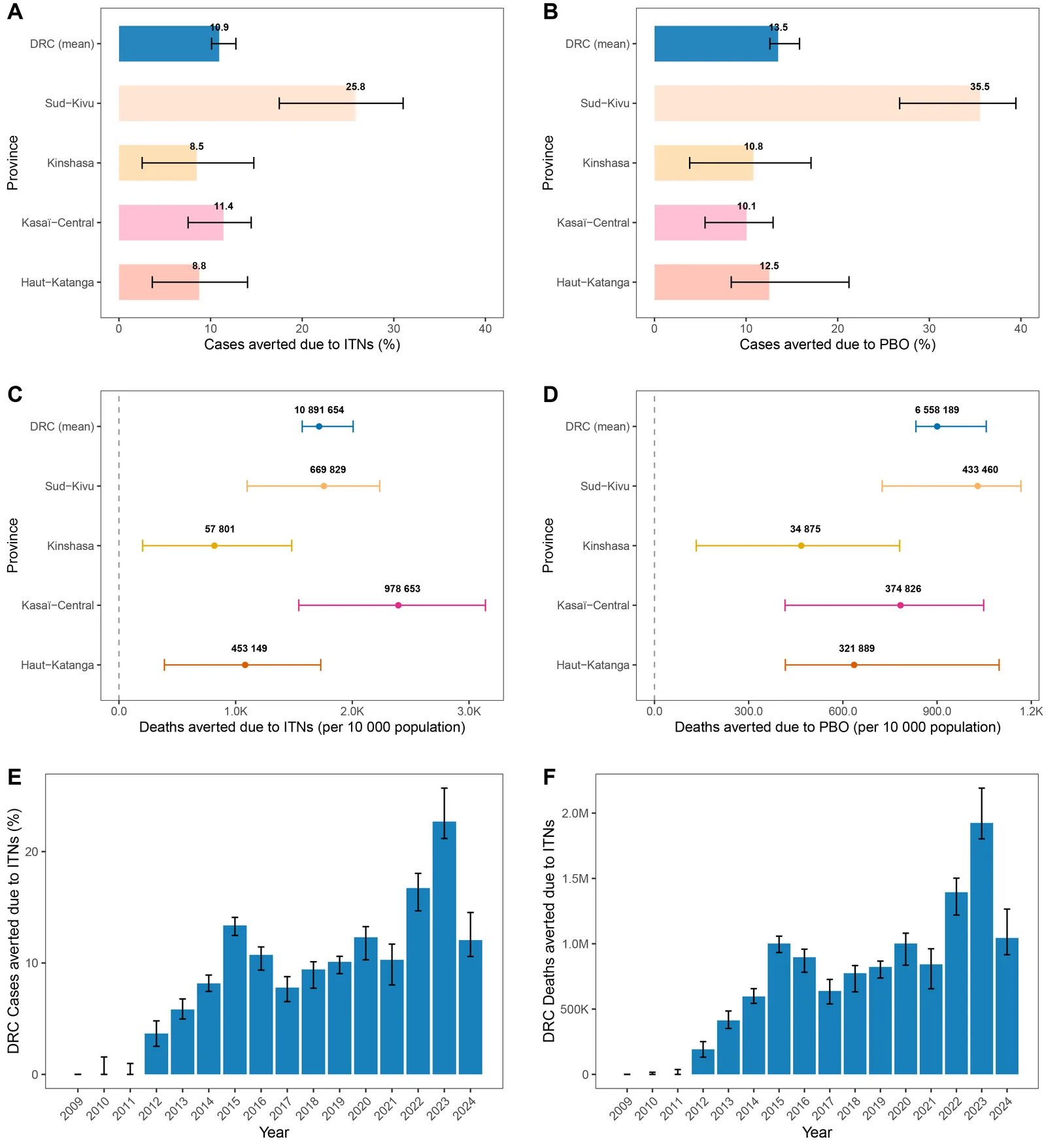
Estimated malaria cases and deaths averted by insecticide-treated nets in the Democratic Republic of the Congo (DRC). Panels **A** and **C** show the overall impact of ITN use during 2009–2024, while panels **B** and **D** show the additional impact of pyrethroid-PBO ITNs during 2019–2024 relative to a pyrethroid-only counterfactual. Estimates are presented nationally and for four selected provinces; estimates for the remaining provinces are provided in Supplementary Figs. S10–S13. Bars and points show posterior medians, with horizontal lines representing 95% credible intervals. **A** Percentage of malaria clinical cases averted by ITNs; numbers beside bars give the corresponding median percentages. **B** Additional percentage of cases averted by pyrethroid-PBO ITNs; numbers beside bars give the corresponding median percentages. **C** Malaria deaths averted per 10,000 population by ITNs; numbers beside points indicate the corresponding cumulative number of deaths averted over 2009–2024. **D** Malaria deaths averted per 10,000 population by pyrethroid-PBO ITNs relative to the pyrethroid-only counterfactual; numbers beside points indicate the corresponding cumulative number of deaths averted over 2019–2024. **E** Annual percentage of malaria clinical cases averted by ITNs. **F** Annual number of malaria deaths averted by ITNs.

The switch from pyrethroid-only ITNs to pyrethroid-PBO ITNs is predicted to have substantially improved malaria prevention. In areas where pyrethroid-PBO ITNs were distributed, the percentage of cases averted by ITNs increased from 5.0% to 35.5%. Nationally, the corresponding increase was approximately 13.5 percentage points. Cumulatively over 2019–2024, pyrethroid-PBO ITNs were estimated to avert approximately 5.73 million additional malaria cases (95% CrI: 3.43–7.82 million) relative to the pyrethroid-only counterfactual. The corresponding mortality impact is shown in Fig. 4D. In 2024, 63.5% of nets in circulation were predicted to be pyrethroid-PBO ITNs, averting an additional 1.41 million cases (95% CrI: 0.95–2.02 million) and 2.72 thousand deaths (95% CrI: 1.83–3.88 thousand) relative to continued use of pyrethroid-only ITNs. Impact varied across the country, with Sud-Kivu again showing the largest modelled reduction (about 35.5%), whereas reductions for Kinshasa and Kasaï-Central were smaller (approximately 10.8% and 10.1%), and Haut-Katanga was around 12.5% (for other provinces, see Supplementary Figs. S10–S13).

### 3.5 Cases and deaths attributable to pyrethroid resistance

Predicted malaria cases and deaths attributable to pyrethroid resistance varied nationally and across the four illustrated provinces. At the national level, approximately 8.6% of malaria cases were attributable to pyrethroid resistance, corresponding to approximately 33.6 million additional cases (95% CrI: 31.5–37.0 million). The model also estimated approximately 12 deaths attributable to resistance per 10,000 population, corresponding to approximately 76,213 deaths nationally. Sud-Kivu showed the largest modelled percentage increase in malaria cases attributable to resistance (approximately 24.2%). Among the four illustrated provinces, Kasaï-Central had the largest absolute number of deaths attributable to resistance, with approximately 5644 deaths over the study period, followed by Haut-Katanga (3837 deaths) and Sud-Kivu (3782 deaths). The corresponding estimate was substantially lower in Kinshasa, at approximately 235 deaths (Fig. 5).

**Fig. 5:**
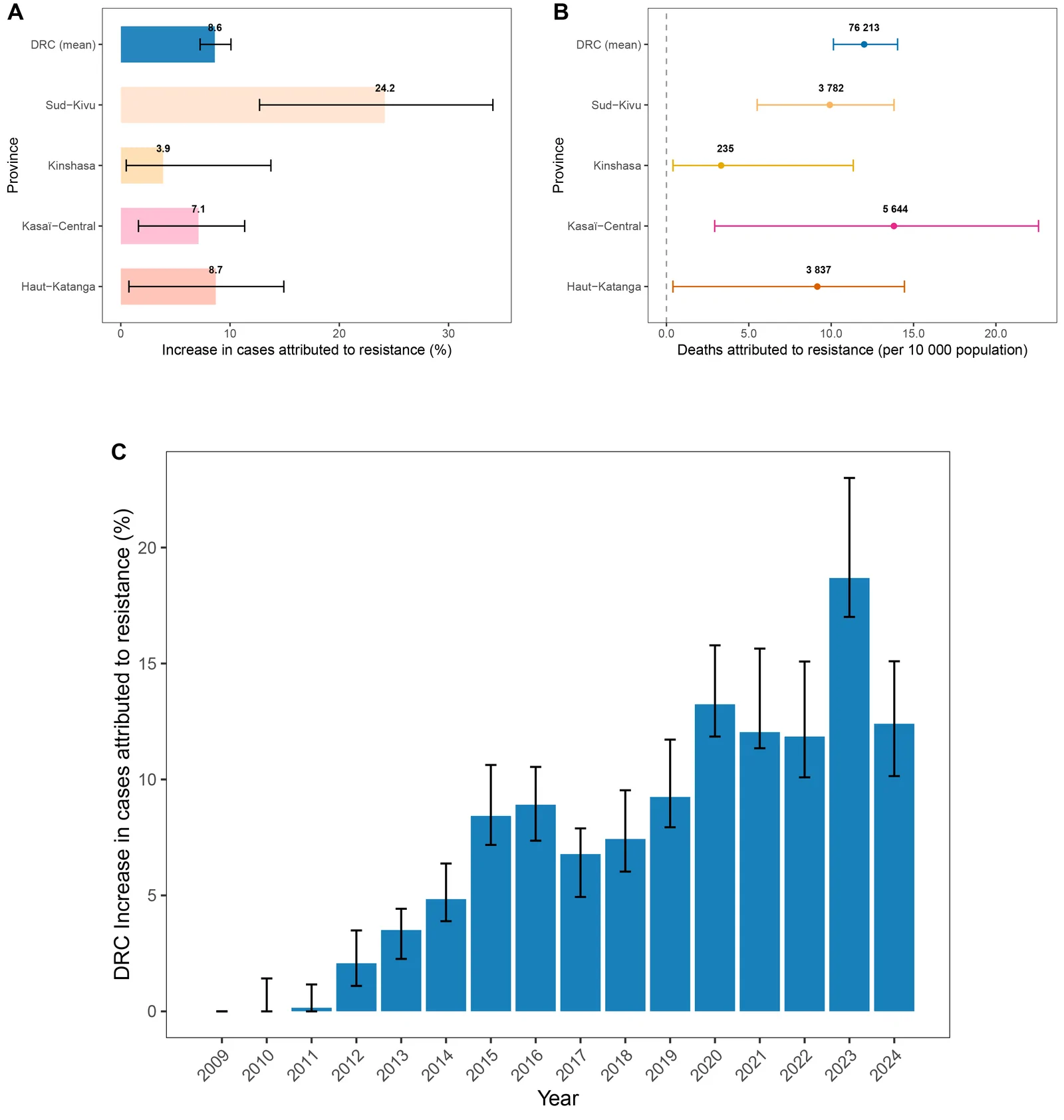
Malaria cases and deaths attributable to pyrethroid resistance. All panels show model-derived estimates of the additional malaria burden attributable to pyrethroid resistance between 2009 and 2024, nationally and for selected provinces. Results for the remaining provinces are provided in Supplementary Figs. S14 and S15. Bars and points show posterior median estimates, with lines indicating 95% credible intervals. **A** Percentage increase in malaria clinical cases attributable to pyrethroid resistance. Percentages beside the bars indicate the corresponding percentage of additional cases attributed to resistance. **B** Malaria deaths attributable to pyrethroid resistance per 10,000 population. Numbers beside the points indicate the corresponding cumulative number of additional deaths attributed to resistance. **C** National time series of the percentage increase in malaria clinical cases attributable to pyrethroid resistance.

The model-derived estimates of the percentage of malaria cases attributed to resistance increased consistently at the national level over time as pyrethroid resistance and ITN use increased. Estimates were less than 5% from 2009 to 2013, about 10% by 2016, and peaked at about 25% by 2023–2024. As resistance grew, the differences between provinces became more pronounced, with uncertainty also increasing in later years. Cumulative province-level estimates of malaria deaths and additional cases attributable to pyrethroid resistance are provided in Supplementary Figs. S14 and S15, respectively.

## 4 Discussion

This analysis combined entomological surveillance data from the Democratic Republic of the Congo with a malaria transmission model to examine the impact of ITNs and the extent to which pyrethroid resistance may have reduced this impact. The model indicates that ITNs prevented substantial numbers of malaria cases and deaths, but that their impact was lower when resistance was included. The inferred resistance trajectory is consistent with earlier regional studies showing that pyrethroid resistance in *Anopheles* populations has increased since the late 2000s (Hemingway et al., 2016; Ranson and Lissenden, 2016). The analysis extends this work by estimating the possible epidemiological consequences of this decline in susceptibility. The overall impact of ITNs was evaluated over 2009–2024, whereas the additional effect of pyrethroid-PBO ITNs was assessed over 2019–2024. The cumulative estimates for these two periods are therefore not directly comparable. The DRC has one of the highest malaria burdens in the world and suffers from chronic underfunding of epidemiological and entomological surveillance. Modelling studies such as this are no substitute for greater investment in health surveillance. Nevertheless, where these data are lacking, modelling can help communicate the benefits of malaria prevention activities to policymakers and non-specialist audiences. The estimates also rely on relationships derived from experimental hut trial data collected elsewhere in Africa. Their transferability to the DRC is uncertain, and the results should therefore be considered alongside the available observations, model assumptions and uncertainty propagated through the analysis.

The discriminating-dose bioassay remains a standardised and widely used method for monitoring insecticide resistance (WHO, 2016), although it provides only a partial indication of the likely effect of resistance and can be subject to substantial measurement error. Variation in bioassay mortality over time and between locations has been reported elsewhere in Africa (Glunt et al., 2014; Hancock et al., 2020), and a similar pattern was observed in the DRC. The observations were sparse and unevenly distributed, with considerable variation between assays conducted in the same year. The beta-binomial model allowed for variation beyond that expected from differences in the number of mosquitoes tested, but the data were insufficient to separate the effects of location, vector population, insecticide, laboratory or assay conditions. Individual observations may therefore differ substantially from the estimated national mean. The broad increase in resistance remained when Kinshasa was excluded and when neighbouring provinces were grouped. However, these analyses were still dominated by the best-sampled provinces: Kinshasa in the Kinshasa-Kongo-Central group and Kasaï-Central in the Kasaï-Central-Kasaï-Oriental-Sankuru group. The national trajectory should therefore be viewed as a summary of the general temporal pattern required by the transmission model rather than as a precise estimate for every province.

Sample-size information was missing for 29 of the 205 bioassays. Although assigning 100 mosquitoes to these observations may have increased the statistical weight given to them, this was the most commonly reported sample size among the remaining assays. Repeating the analysis using assumed sample sizes of 50, 83 and 100 mosquitoes produced similar trajectories and credible intervals, indicating that this assumption had little influence on the broad national trend supplied to the transmission model. A total of 10 of the 26 provinces had no bioassay data over the 16-year period. These provinces contained approximately 30.9 million people in 2024, equivalent to approximately 29.2% of the national population. It is unclear whether unpublished data exist and are being used to support decisions on ITN deployment. Future surveillance efforts should prioritise broader geographical coverage, particularly in provinces with few or no observations. This should be accompanied by repeated sampling through a consistent network of sentinel sites. More intensive surveillance may also be useful in high-burden areas or locations where resistance appears to be changing rapidly, but it should not come at the expense of broader national coverage. More representative data would allow province-specific trends to be estimated and could help identify where more expensive next-generation ITNs are likely to provide the greatest benefit.

To our knowledge, there are no experimental hut trial (EHT) data from the DRC (Nash et al., 2021). We used previously published relationships between bioassay mortality and experimental hut trial outcomes to parameterise the model. The equations and their parameterisation are described in Supplementary Text S1 (Supporting Methods). Although these relationships have been assessed in several African settings (Sherrard-Smith et al., 2022b), their application to the DRC assumes broadly similar vector species composition and ecological conditions (Sinka et al., 2010; Coetzee et al., 2013). Although models using these relationships have reproduced differences in ITN efficacy observed in Benin, Tanzania and Uganda, they cannot represent every consequence of resistance. Delayed mortality, changes in mosquito behaviour, variation in vector ecology, and the underlying resistance mechanisms may all alter the epidemiological effect (Mulamba et al., 2014; Viana et al., 2016; Walker et al., 2016). Resistance was used to determine the initial deterrent, killing, and blood-feeding effects assigned to each cohort of pyrethroid-only ITNs at the time of distribution. Efficacy then declined as the nets aged, but it was not recalculated when resistance changed between campaigns. The model may therefore underestimate the effect of resistance if susceptibility declines substantially while previously distributed nets remain in use. We are unaware of any empirical evidence showing how ITN use changed between 2008 and 2024. In the absence of such data, we used estimates derived from multi-country analyses (Glover et al., 2026), constrained by reported campaign timing and the available survey data from the DRC. The timing of increases following campaigns and subsequent declines therefore reflects the fitted usage model rather than detailed observations from each province. Net retention may vary considerably between settings (Solomon et al., 2018; Obi et al., 2020; Diouf et al., 2022), but the available data were insufficient to estimate this variation.

The model estimated that national ITN use rarely exceeded 50%, suggesting that improved delivery, more frequent campaigns or stronger continuous-distribution systems could further reduce malaria. This estimate remains uncertain because ITN use was assumed to decline at a constant rate following each campaign. Differences in access, retention, replacement, durability and use could produce a different temporal pattern. Uncertainty in ITN use may also contribute to the disagreement between modelled and observed prevalence, although comparisons between the resistance and no-resistance scenarios are less affected because the same ITN-use trajectory was applied to both.

The analysis linked entomological, intervention, demographic and epidemiological data collected at different times, in different locations and at different spatial scales. Differences in sampling, laboratory procedures and reporting completeness could not all be represented. The outputs are therefore more informative as comparisons between scenarios than as precise estimates for a particular province or year. The model did not closely reproduce the DHS malaria-prevalence estimates for 2013–2014 and 2023–2024. These observations were not used to fit the model and were included only as an external comparison. The projections should therefore be interpreted mainly in terms of the relative difference between the resistance and no-resistance scenarios rather than as precise predictions for either survey period. The disagreement may reflect uncertainty in both the reconstructed ITN-use trajectory and the estimated resistance trajectory, as well as geographical variation obscured by the national-level model. Prevalence increased between the surveys in some provinces, including Haut-Katanga and Sud-Kivu, but declined in others.

Prevalence was generally four to seven percentage points higher when resistance was included. This difference was relatively modest because transmission was already high and resistance reduced, but did not remove, the protection provided by pyrethroid-only ITNs. The relationship between transmission intensity and cross-sectional prevalence is also nonlinear. Cases and deaths accumulate over time in the large population of the DRC, so a small but persistent difference in transmission can produce substantial cumulative effects even when the prevalence trajectories remain close. The same year-specific calibration factors were applied to the resistance and no-resistance scenarios, so the difference between them represents an internal counterfactual comparison rather than an estimate derived from the two DHS surveys. Estimates of deaths averted and deaths attributable to resistance also relied on case-fatality ratios derived from WHO data, which may be affected by incomplete diagnosis, under-reporting and unequal access to treatment (Gething et al., 2012). The absolute numbers should therefore be interpreted cautiously, although the comparison consistently indicates that resistance reduced the effectiveness of pyrethroid-only ITNs. Within the simulations, pyrethroid-PBO ITNs reduced the adverse effect of resistance relative to the continued use of pyrethroid-only ITNs, although prevalence remained higher than in the fully susceptible counterfactual scenario. These findings describe relative differences between modelled scenarios and should not be treated as precise predictions for individual provinces. The analysis did not include ITNs containing other active ingredients, so no conclusions can be drawn about the potential impact of those products.

Several additional limitations warrant emphasis. We assumed that underlying malaria prevalence remained stable over the study period, although transmission may have changed because of urbanisation, climate variability or changes in water management. Apart from changes in the case-fatality ratio, treatment efficacy and case management were held constant because suitable time-varying data were unavailable. Changes in malaria policy and the emergence of antimalarial drug resistance may also have affected transmission during the study period, but these were not included in the model (Ashley et al., 2014; Uwimana et al., 2020). We also assumed that mosquito species composition remained unchanged. This may not hold throughout the DRC because mosquito species differ in their feeding behaviour, ecology, and susceptibility to insecticides, and these differences may alter the effects of ITNs and resistance (Sinka et al., 2010; Mulamba et al., 2014). Lastly, we assumed that the patterns and duration of ITN use were similar across regions and net products, although behaviour, cultural practices and ITN durability may have varied across the DRC and over the study period. If these factors varied substantially by setting or product type, some of the results could differ.

Despite these limitations, the analysis supports the continued importance of ITNs for malaria prevention in the DRC and indicates that pyrethroid resistance has probably reduced the protection provided by pyrethroid-only ITNs. Pyrethroid-PBO ITNs restored some of this lost protection in the model, although the magnitude and geographical distribution of their additional benefit remain uncertain. Better subnational predictions will require broader entomological surveillance, improved information on ITN access and use, and local evidence linking resistance measurements to ITN performance. When interpreted cautiously, modelling analyses such as this can support malaria-control planning and strengthen the case for further investment in surveillance and vector control in the DRC.

## 5 Conclusions

ITNs remain important for malaria prevention in the DRC, but the model indicates that increasing pyrethroid resistance has reduced the protection provided by pyrethroid-only ITNs. Pyrethroid-PBO ITNs restored some of this lost protection in the simulations, although the magnitude and geographical distribution of their additional benefit remain uncertain because resistance surveillance and epidemiological observations were sparse and unevenly distributed. Broader entomological surveillance, improved information on ITN access and use, and local evidence linking resistance measurements to ITN performance will be needed to improve subnational estimates and inform vector-control planning in the DRC.

## CRediT authorship contribution statement

**Chadrac Ntembue Tshiakuisha:** Conceptualisation, Methodology, Software, Formal analysis, Investigation, Visualisation, Data curation, Writing - original draft, Writing - review & editing. **Andrew C. Glover:** Methodology, Data curation, Formal analysis, Writing - review & editing, Resources. **Francis Watsenga:** Data curation, Project administration, Conceptualisation, Writing - review & editing, Resources. **Charles S. Wondji:** Data curation, Project administration, Conceptualisation, Resources, Writing - review & editing, Funding acquisition. **Samuel Bowong:** Conceptualisation, Methodology, Writing - review & editing, Supervision. **Thomas S. Churcher:** Conceptualisation, Resources, Methodology, Data curation, Writing - original draft, Writing - review & editing, Supervision.

## Statement on the use of AI-assisted technologies

During the preparation of this work, the corresponding author used ChatGPT (OpenAI) to assist with language editing and presentation of the manuscript. After using this tool, the authors reviewed and edited the content as needed and take full responsibility for the content of the published article.

## Funding

This research was supported by the 10.13039/100000865Bill & Melinda Gates Foundation through the AComVec Project (INV-047049). Andrew C. Glover and Thomas S. Churcher acknowledge funding from the 10.13039/501100000265Medical Research Council (MRC) Centre for Global Infectious Disease Analysis (MR/R015600/1), jointly funded by the 10.13039/501100000265MRC and the 10.13039/501100020171UK Foreign, Commonwealth and Development Office (FCDO) under the 10.13039/501100000265MRC/10.13039/501100020171FCDO Concordat agreement. This work is also part of the EDCTP2 programme supported by the 10.13039/501100000780European Union.

## Declaration of competing interests

The authors declare that they have no known competing financial interests or personal relationships that could have appeared to influence the work reported in this paper.

## Data Availability

The data supporting the conclusions of this article are included within the article and its supplementary file. The data and code used to reproduce the analyses and results presented in this article are publicly available in the following GitHub repository: https://github.com/Bright1964/Chadrac-Ntembue-code-.git.
