# Supplementary material for: The spread of pyrethroid-resistant mosquitoes and their predicted malaria burden across the Democratic Republic of the Congo
Source: Curr Res Parasitol Vector Borne Dis. 2026 Sep 19;10:100439. doi: 10.1016/j.crpvbd.2026.100439 (PMC13633922; doi:10.1016/j.crpvbd.2026.100439)
Supplement: Supplementary file 1 [file mmc1.pdf]

Supplementary Material

The spread of pyrethroid-resistant mosquitoes and their predicted malaria burden across the Democratic Republic of the Congo

Chadrac Ntembue Tshiakuisha[a,b,d,*], Andrew C. Glover[c], Francis Watsenga[d]
Charles S. Wondji[a,e], Samuel Bowong[b,f], Thomas S. Churcher[c]

[a]Centre for Research in Infectious Diseases, Yaoundé, Cameroon.
[b]University of Douala, Department of Mathematics and Computer Science, Douala, Cameroon.
[c]MRC Centre for Global Infectious Disease Analysis, Imperial College London, London, UK.
[d]Centre Entomologique de Formation, Recherche et Expertise de Kimpese, Kimpese, DRC.
[e]Department of Vector Biology, Liverpool School of Tropical Medicine, Liverpool, UK.
[f]IRD, UMMISCO, Sorbonne University, Bondy, France.

*Corresponding author: Chadrac Ntembue Tshiakuisha, chadrac@aims.edu.gh

Supplementary Text S1. Supporting Methods

A.1 Sensitivity analysis for missing bioassay sample sizes

The number of mosquitoes tested was not reported for 29 of the 205 bioassays included in the analysis, corresponding to 14.1% of the dataset. In the primary analysis, these observations were assigned a sample size of 100 mosquitoes, corresponding to four replicates of 25 mosquitoes under the standard WHO discriminating-dose bioassay protocol. Among the 176 bioassays for which sample-size information was available, 109 assays (61.9%) tested exactly 100 mosquitoes. For observations with missing sample-size information, the number of surviving mosquitoes was reconstructed from the reported percentage mortality and the assumed number tested. Reported sample sizes were retained for all other observations. The influence of this assumption was assessed by repeating the complete national resistance analysis using assumed sample sizes of 50, 83 and 100 mosquitoes for the 29 affected bioassays. A sample size of 50 provided a more conservative weighting and was consistent with the approach used by Nash et al. (2021). The value of 83 corresponded approximately to the mean sample size across the complete assembled dataset after assigning 100 mosquitoes to observations with missing sample-size information. The model structure, prior distributions and Markov chain Monte Carlo settings were unchanged across the three analyses.

A.2 Geographical sensitivity analyses of resistance

Sampling locations were retained where available, but observations were sparse and unevenly distributed across sites and provinces. The available data were therefore insufficient to support reliable province-specific temporal resistance trajectories. To examine whether the national trajectory was disproportionately influenced by observations from Kinshasa, the resistance model was fitted after excluding all observations from Kinshasa. A separate model was also fitted using Kinshasa observations alone. Exploratory analyses were additionally conducted for two groups of neighbouring provinces: Kinshasa with Kongo-Central, and Kasaï-Central with Kasaï-Oriental and Sankuru. The same beta-binomial likelihood, generalised logistic temporal function, prior distributions and HMC settings used in the national analysis were retained in each sensitivity analysis. The grouped analyses were treated as sensitivity checks rather than reliable local trajectories because observations remained concentrated in the best-sampled province within each group.

A.3 Posterior predictive assessment of the resistance model

Model adequacy was assessed using posterior predictive checks. For each retained posterior draw, replicate bioassay observations were generated from the beta-binomial model using the original observation years and sample sizes. The distribution of these replicated observations was compared with the observed data to assess whether the model reproduced the overall degree of variation between bioassays. The 95% credible interval around $m(t)$ represents uncertainty in the estimated national mean survival trajectory. This interval is narrower than the posterior predictive distribution, which represents the wider variation expected for an individual bioassay.

A.4 Converting bioassay survival into ITN entomological effects

This analysis was conducted separately from the model used to estimate the national resistance trajectory. No additional model was fitted to the DRC bioassay observations at this stage. Instead, previously published relationships were used to translate discriminating-dose bioassay survival into the entomological outcomes expected when a mosquito encounters an ITN during a feeding attempt (Nash et al., 2021; Churcher et al., 2024). Bioassays provide a standardised measure of pyrethroid resistance under controlled laboratory conditions, whereas experimental hut trials measure mosquito entry, blood-feeding, survival, and mortality following exposure to an ITN under more field-relevant conditions. Let $q \in \{\mathrm{PY}, \mathrm{PBO}\}$ denote the ITN type, where PY represents a pyrethroid-only ITN and PBO represents a pyrethroid–PBO ITN. Let k index the ITN distribution round and y_k denote the corresponding year. For each posterior simulation, the sampled national resistance trajectory was evaluated at y_k, giving the campaign-specific discriminating-dose bioassay survival probability

$$m_k = m(y_k). \tag{1}$$

For a newly distributed pyrethroid-only ITN, the corresponding probability that a mosquito survived after entering an experimental hut was calculated using the relationship reported by Nash et al. (2021):

$$h_{\mathrm{PY},k} = 1 - \left(\frac{1 - m_k}{\beta}\right)^{-\alpha}, \tag{2}$$

where $h_{\mathrm{PY},k}$ is the probability of survival conditional on entry into a hut containing a pyrethroid-only ITN. The corresponding probability of mortality after hut entry was

$$\ell_{\mathrm{PY},k} = 1 - h_{\mathrm{PY},k}. \tag{3}$$

The parameters α and β determine the shape of the relationship between discriminating-dose bioassay survival and experimental-hut survival. Uncertainty in this conversion was propagated using the distributions

$$\alpha \sim \mathcal{N}(0, 2^2), \qquad \beta \sim \mathcal{N}(0,1). \tag{4}$$

For pyrethroid–PBO ITNs, mortality was obtained from the published relationship linking mortality in huts containing pyrethroid-only and pyrethroid–PBO ITNs (Churcher et al., 2024). This can be written compactly as

$$\ell_{\mathrm{PBO},k} = B_{\mathrm{PBO}}(\ell_{\mathrm{PY},k}, \boldsymbol{\phi}_{\mathrm{PBO}}), \qquad h_{\mathrm{PBO},k} = 1 - \ell_{\mathrm{PBO},k}, \tag{5}$$

where $B_{\mathrm{PBO}}(\cdot)$ denotes the published transformation and $\boldsymbol{\phi}_{\mathrm{PBO}}$ contains its associated parameters. More generally, for either net type,

$$h_{q,k} = H_q[m_k, \boldsymbol{\psi}_q], \qquad \ell_{q,k} = 1 - h_{q,k}, \tag{6}$$

where $H_q(\cdot)$ summarises the net-type-specific conversion from bioassay survival to experimental-hut survival. Both $h_{q,k}$ and $\ell_{q,k}$ are conditional on the mosquito entering the experimental hut.

A.5 Deterrence before hut entry

Deterrence was modelled separately from mortality and blood-feeding among mosquitoes that entered the hut. Let $D_{q,k}$ denote the probability that a mosquito was deterred before entering a hut containing a newly distributed net of type q. Following the published experimental-hut relationship,

$$D_{q,k} = \delta_1 \exp\left[\frac{\delta_2\{1 - \exp(\delta_3 h_{q,k})\}}{\delta_3}\right]. \tag{7}$$

The probability that a mosquito was not deterred and entered the treatment hut was therefore

$$A_{q,k} = 1 - D_{q,k}. \tag{8}$$

In the experimental-hut data used to estimate this relationship, $A_{q,k}$ corresponds to the relative mosquito entry into a treatment hut compared with a control hut containing an untreated net:

$$\hat{A}_{q,k} = \frac{N_{q,k}^{\text{treated}}}{N_k^{\text{control}}}, \tag{9}$$

where $N_{q,k}^{\text{treated}}$ and N_k^{control} are the numbers of mosquitoes collected in the treatment and control huts, respectively. Thus, $1 - D_{q,k}$ represents relative hut entry and not mosquito survival or mortality.

A.6 Outcomes conditional on hut entry

Among mosquitoes entering the hut, three mutually exclusive outcomes were considered: exiting without blood-feeding, successfully blood-feeding and surviving, or dying. Let $E_{q,k}$, $F_{q,k}$, and $M_{q,k}$ denote these respective probabilities. Mortality conditional on hut entry was

$$M_{q,k} = \ell_{q,k}. \tag{10}$$

The probability of successfully blood-feeding and surviving after entering the hut was calculated as

$$F_{q,k} = 1 - \exp\left[\frac{\delta_4\{1 - \exp(\delta_5 h_{q,k})\}}{\delta_5}\right]. \tag{11}$$

The probability of entering the hut but exiting without feeding was obtained as the remaining conditional outcome:

$$E_{q,k} = 1 - F_{q,k} - M_{q,k}. \tag{12}$$

Consequently,

$$E_{q,k} + F_{q,k} + M_{q,k} = 1. \tag{13}$$

A.7 Outcomes for a complete mosquito feeding attempt

Deterrence was then combined with the outcomes conditional on hut entry. The probabilities of failing to feed, successfully feeding, and dying during the complete feeding attempt were

$$\begin{aligned} E_{q,k}{}' &= D_{q,k} + A_{q,k} E_{q,k}, \\ F_{q,k}{}' &= A_{q,k} F_{q,k}, \\ M_{q,k}{}' &= A_{q,k} M_{q,k}. \end{aligned} \tag{14}$$

The quantity $E_{q,k}{}'$ includes both mosquitoes deterred before entering the hut and mosquitoes that entered but exited without feeding. The quantities $F_{q,k}{}'$ and $M_{q,k}{}'$ represent successful blood-feeding and mortality, respectively, after accounting for deterrence. These outcomes satisfy

$$E_{q,k}{}' + F_{q,k}{}' + M_{q,k}{}' = 1. \tag{15}$$

Let k_0 denote the probability that a mosquito successfully blood-feeds in the absence of an intervention. In the transmission model, this parameter was fixed at $k_0 = 0.699$, following the parameterisation of the published ITN efficacy model (Nash et al., 2021; Churcher et al., 2024). The initial entomological parameters assigned to the ITN cohort distributed during round k were

$$\begin{pmatrix} r_{n0,k}^{(q)} \\ s_{n0,k}^{(q)} \\ d_{n0,k}^{(q)} \end{pmatrix} = g_q[m_k, \boldsymbol{\eta}_q] = \begin{pmatrix} \left(1 - \frac{F_{q,k}{}'}{k_0}\right)\left(\frac{E_{q,k}{}'}{E_{q,k}{}' + M_{q,k}{}'}\right) \\ \frac{F_{q,k}{}'}{k_0} \\ \left(1 - \frac{F_{q,k}{}'}{k_0}\right)\left(\frac{M_{q,k}{}'}{E_{q,k}{}' + M_{q,k}{}'}\right) \end{pmatrix}. \tag{16}$$

Here, $r_{n0,k}^{(q)}$ is the initial probability that a mosquito fails to feed and repeats its feeding attempt, either because it was deterred or because it entered the hut and exited unfed. The quantity $s_{n0,k}^{(q)}$ is the initial probability of successful blood-feeding, and $d_{n0,k}^{(q)}$ is the initial probability of mosquito mortality. The subscript $n0$ denotes the effect of a newly distributed net. These three mutually exclusive probabilities satisfy

$$r_{n0,k}^{(q)} + s_{n0,k}^{(q)} + d_{n0,k}^{(q)} = 1. \tag{17}$$

The parameter vector $\boldsymbol{\eta}_q$ contains the parameters used in the bioassay-to-hut conversion and in the relationships describing deterrence and successful blood-feeding. Accordingly, $g_q(\cdot)$ is shorthand for the deterministic sequence of transformations described above and does not represent an additional model fitted to the DRC bioassay observations. For each transmission-model simulation, uncertainty in the resistance trajectory and in the published bioassay-to-hut relationships was propagated jointly when calculating $r_{n0,k}^{(q)}$, $s_{n0,k}^{(q)}$, and $d_{n0,k}^{(q)}$. These campaign-specific values were passed to `set_bednets()` when each new ITN cohort was introduced into the transmission model. Nets distributed in different years could therefore have different initial efficacies, reflecting the estimated resistance level at the time of deployment. Following distribution, the transmission model was updated at daily time steps and accounted for both the loss of nets from use and the decline in their insecticidal efficacy as they aged. Subsequent changes in resistance did not modify the efficacy parameters of nets that were already in use. Resistance therefore determined the initial efficacy of each newly distributed ITN cohort rather than continuously modifying the efficacy of existing nets between campaigns. The relationship between bioassay survival and experimental-hut survival, and the resulting mosquito outcomes, are shown in Supplementary Fig. S3.

Supplementary Table S1. Initial entomological efficacy parameters for pyrethroid-only and pyrethroid-PBO ITNs.

Parameter	Biological description	Mean	95% CrI
**Pyrethroid-only ITNs ($q = \mathrm{PY}$)**			
$d_{n0,k}^{(\mathrm{PY})}$	Initial probability that a mosquito dies during a feeding attempt on a newly deployed pyrethroid-only ITN	0.260	[0.244, 0.277]
$r_{n0,k}^{(\mathrm{PY})}$	Initial probability that a mosquito is repelled during a feeding attempt on a newly deployed pyrethroid-only ITN	0.731	[0.716, 0.746]
$s_{n0,k}^{(\mathrm{PY})}$	Initial probability that a mosquito successfully blood-feeds during a feeding attempt on a newly deployed pyrethroid-only ITN	0.009	[0.007, 0.010]
**Pyrethroid-PBO ITNs ($q = \mathrm{PBO}$)**			
$d_{n0,k}^{(\mathrm{PBO})}$	Initial probability that a mosquito dies during a feeding attempt on a newly deployed pyrethroid-PBO ITN	0.375	[0.367, 0.382]
$r_{n0,k}^{(\mathrm{PBO})}$	Initial probability that a mosquito is repelled during a feeding attempt on a newly deployed pyrethroid-PBO ITN	0.624	[0.617, 0.632]
$s_{n0,k}^{(\mathrm{PBO})}$	Initial probability that a mosquito successfully blood-feeds during a feeding attempt on a newly deployed pyrethroid-PBO ITN	0.001	[0.001, 0.001]

A.8 Additional details of the malaria transmission model

The malaria transmission framework is a non-spatial, individual-based model of *Plasmodium falciparum* transmission in humans coupled to a mosquito population model. Individuals are characterised by age, infection status, clinical disease and acquired immunity. Age-dependent biting patterns result in lower mosquito exposure among younger individuals, while repeated exposure contributes to the development of anti-parasite and clinical immunity. Human infection and disease events are simulated together with mosquito population and intervention dynamics. Further details are available in the original model publications (Griffin et al., 2010; White et al., 2011).

Baseline transmission intensity was estimated separately for each province. Provincial entomological inoculation rates were selected to reproduce malaria prevalence before the introduction of the modelled intervention history, using prevalence and associated covariates obtained through the foresite package from Malaria Atlas Project estimates. Provincial inputs included malaria prevalence, mosquito species composition, intervention coverage, rainfall and demographic characteristics. Province-specific mosquito species composition was treated as a fixed model input and was not allowed to vary over time.

Because resistance observations were sparse and unevenly distributed across provinces, including years with no surveillance, the same sampled national resistance trajectory was used for all provinces. All other transmission, demographic and intervention inputs were specified at the provincial level. Provincial prevalence estimates were aggregated to the national level using population-weighted means, whereas provincial numbers of cases and deaths were aggregated by summation.

A.9 Propagation of parameter uncertainty

One hundred posterior simulations were conducted for each province and intervention scenario. For each simulation, one complete trajectory was sampled from the posterior distribution of the resistance model and one posterior draw was sampled from the ITN-use model, retaining the correlations between parameters within each fitted model. Posterior uncertainty in the bioassay-to-hut relationships was also sampled when calculating the initial effects assigned to each ITN cohort. The sampled resistance trajectory, ITN-use parameters, and

bioassay-to-hut parameters were then propagated together through the malaria transmission simulations. Posterior medians and 95% credible intervals were calculated from the resulting distribution of epidemiological outcomes.

A.10 Additional model-checking procedures

Three forms of model checking were undertaken. First, posterior predictive checks were used to assess whether the beta-binomial resistance model reproduced the overall variability observed between bioassays. Second, reconstructed provincial ITN-use trajectories were compared with available DHS estimates of the proportion of individuals who slept under an ITN the previous night. Third, model-derived malaria prevalence was compared with prevalence estimates from the 2013–2014 and 2023–2024 DHS surveys. The DHS prevalence estimates were not used to fit the transmission model. They were treated as an external assessment of consistency with independent observations rather than as formal model validation.

A.11 Changes in ITN use over time

The model used to reconstruct ITN use over time was separate from both the national resistance model and the bioassay-to-hut conversion. It was not refitted using HMC in the present analysis. Instead, posterior parameter estimates were taken from the mixed-effects analysis of household-survey data reported by Glover et al. (2026). ITN mass campaigns by province were collated from National Malaria Control Programme (NMCP) reports from 2009 to 2024. There is very limited information on the percentage of people using ITNs over time across the 26 provinces of the DRC. Instead, we used a recent continent-wide meta-analysis that estimated ITN use immediately after a campaign and the average duration of net use by fitting mixed-effects models to DHS household survey data reporting the proportion of individuals who slept under an ITN the previous night (Glover et al., 2026). Following this approach, ITNs were assumed to be lost from use at a constant rate after distribution, and ITN use was assumed to be uniform across age and sex groups. The reconstructed trajectories were compared with empirical ITN-use estimates from the 2013–2014 and 2023–2024 DHS surveys. The earlier survey reported estimates for 11 former administrative regions. These estimates were mapped to the current provincial structure using the administrative subdivisions through which the former regions were divided into the present 26 provinces.

Let $p_r(t)$ denote the probability of ITN use in province r at time t. Following Glover et al. (2026), total ITN use was represented as the sum of contributions from mass campaigns and continuous distribution channels:

$$p_r(t) = c_r(t) + q_r(t) \qquad (18)$$

where $c_r(t)$ denotes use attributable to campaign-distributed ITNs and $q_r(t)$ denotes use attributable to ITNs obtained through continuous distribution channels.

Let $u_r(t)$ denote the underlying expected level of ITN use in province r at time t. The contribution from campaign-distributed ITNs was represented as

$$c_r(t) = \omega u_r(t) \exp\left[-\frac{\delta_r(t)}{\tau}\right] \qquad (19)$$

where $\delta_r(t)$ is the number of months since the most recent mass-distribution campaign in province r, ω is the proportion of immediate post-campaign use attributable to campaign-distributed nets, and τ is the mean duration over which campaign-derived ITN use declines.

The contribution from continuous distribution channels was given by

$$q_r(t) = (1 - \omega) u_r(t) \qquad (20)$$

The underlying expected use level was described by

$$u_r(t) = u_{\max} \text{logit}^{-1}(\alpha_r + \gamma_r t) \qquad (21)$$

where $u_{\max}$ is the upper limit on ITN use, α_r determines the timing of the increase in ITN use in province r, and γ_r governs the rate of change over time. The decay rate may equivalently be written as $\lambda = 1/\tau$.

Provincial campaign records were used to identify the timing of mass distribution campaigns and to calculate $\delta_r(t)$. The numbers of ITNs distributed during individual campaigns were not used to estimate separate initial use probabilities for each campaign. Model parameters were obtained from the continent-wide meta-analysis described by Glover et al. (2026). For each transmission-model simulation, one joint posterior draw was taken from the mixed-effects ITN-use analysis, preserving the estimated correlations between the campaign contribution, continuous-distribution contribution and decay duration. The resulting ITN-use trajectory was propagated through the transmission model. We considered the period from 2009 to 2024. All ITNs distributed before 2019 were assumed to be pyrethroid-only ITNs, while the proportion of pyrethroid-PBO ITNs distributed from 2019 to 2024 was obtained from the Alliance for Malaria Prevention.

A.12 Epidemiological impact measures

Cases averted by ITNs were calculated by comparing the no-ITN scenario with the reference scenario:

$$A^{\text{ITN}}_{r,t,d} = C^{\text{cal}}_{r,\text{No ITN},t,d} - C^{\text{cal}}_{r,\text{ref},t,d}. \quad (22)$$

Deaths averted by ITNs were defined analogously:

$$A^{\text{ITN,death}}_{r,t,d} = D^{\text{cal}}_{r,\text{No ITN},t,d} - D^{\text{cal}}_{r,\text{ref},t,d}. \quad (23)$$

Cases attributed to pyrethroid resistance were calculated as the difference between the reference and no-resistance scenarios:

$$B^{\text{res}}_{r,t,d} = C^{\text{cal}}_{r,\text{ref},t,d} - C^{\text{cal}}_{r,\text{No resistance},t,d}. \quad (24)$$

with deaths attributed to resistance calculated as

$$B^{\text{res,death}}_{r,t,d} = D^{\text{cal}}_{r,\text{ref},t,d} - D^{\text{cal}}_{r,\text{No resistance},t,d}. \quad (25)$$

The additional cases averted by pyrethroid–PBO ITNs were calculated by comparing the pyrethroid-only counterfactual with the reference scenario:

$$A^{\text{PBO}}_{r,t,d} = C^{\text{cal}}_{r,\text{Pyrethroid only},t,d} - C^{\text{cal}}_{r,\text{ref},t,d}. \quad (26)$$

and the corresponding additional deaths averted were

$$A^{\text{PBO,death}}_{r,t,d} = D^{\text{cal}}_{r,\text{Pyrethroid only},t,d} - D^{\text{cal}}_{r,\text{ref},t,d}. \quad (27)$$

The effects of ITNs and pyrethroid resistance were accumulated over 2009–2024. The additional effects of pyrethroid–PBO ITNs were accumulated over 2019–2024, corresponding to the period in which these nets were included in the model. Results were summarised using the posterior median and 95% credible interval across simulation draws. Mortality outcomes presented per 10,000 population were calculated using the corresponding mean provincial or national population over the relevant analysis period.

B. DRC data

Supplementary Table S2. Summary of insecticide-resistance bioassay data used to estimate temporal changes in pyrethroid resistance in the Democratic Republic of the Congo.

Dataset characteristic	Summary
Country	Democratic Republic of the Congo
Study period	2009–2021
Bioassay records	205
Provinces represented	16
Sampling sites represented	31
Mosquitoes tested	16,934
Bioassay method	WHO discriminating-dose tube bioassay
Outcome used in the resistance model	Mosquito survival 24 h after exposure
Bioassays with reported sample size	176/205 (85.9%)
Bioassays with missing sample size	29/205 (14.1%)
Bioassays testing exactly 100 mosquitoes[a]	109/176 (61.9%)
Assumed sample size for missing observations	100 mosquitoes
Alternative sample sizes used in sensitivity analysis	50 and 83 mosquitoes
Mosquito taxa included	All tested *Anopheles* mosquitoes

B.1 Sensitivity of the resistance trajectory to the geographical distribution of bioassay data

A substantial proportion of the available bioassay observations were collected in Kinshasa. We therefore repeated the analysis using several geographical subsets of the data to assess whether the national resistance trajectory was being driven primarily by observations from the capital. The model was first fitted after removing Kinshasa from the national dataset. Separate models were then fitted to observations from Kinshasa alone, Kinshasa together with Kongo-Central, and a group comprising Kasaï-Central, Kasaï-Oriental and Sankuru (Figure S1).

Removing Kinshasa did not alter the overall conclusion that mosquito survival following exposure to a discriminating dose of pyrethroid insecticide increased over time. The trajectory estimated without Kinshasa remained similar in shape to that obtained from the complete national dataset, although uncertainty increased because fewer observations were available. The Kinshasa-only analysis also indicated an increase in survival, but the observations were highly variable, particularly in the later years.

The trajectories estimated from Kinshasa and Kongo-Central, and from Kasaï-Central, Kasaï-Oriental and Sankuru, showed the same broad temporal pattern. However, there was substantial variation between individual bioassays, reflected by the wide posterior predictive intervals. These results suggest that the increase in pyrethroid resistance was not solely determined by the large number of observations from Kinshasa. Nevertheless, the limited number of observations and years represented in some provincial groups means that these subgroup trajectories should be interpreted as sensitivity analyses rather than precise estimates of province-specific resistance trends.

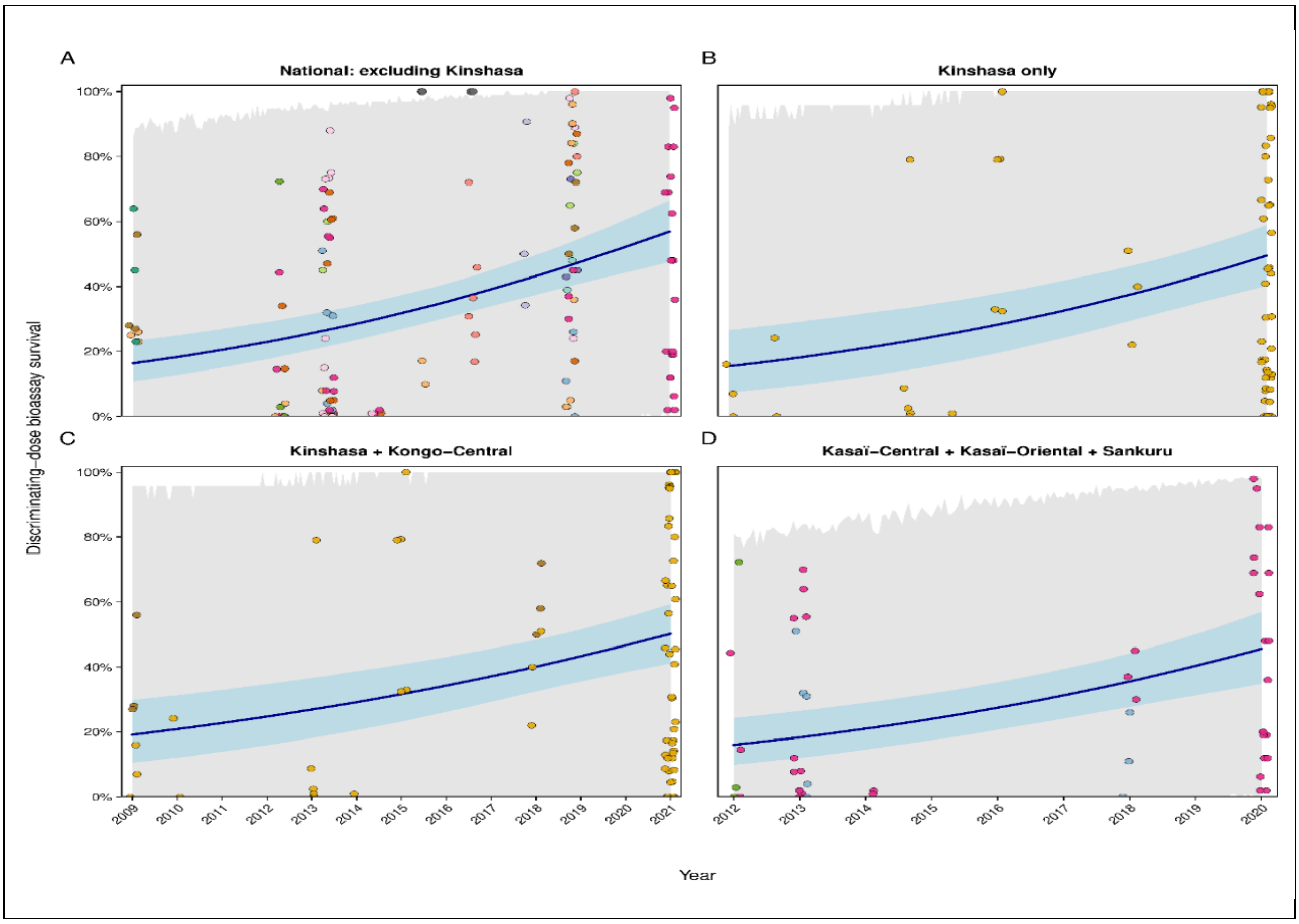

Supplementary Figure S1. Sensitivity of the estimated pyrethroid-resistance trajectory to the geographical distribution of bioassay observations. Panel **A** shows the national resistance trajectory after excluding all observations collected in Kinshasa. Panel **B** shows the trajectory estimated using observations from Kinshasa alone. Panel **C** combines observations from Kinshasa and Kongo-Central, while panel **D** combines observations from Kasaï-Central, Kasaï-Oriental and Sankuru. Points represent the observed proportion of mosquitoes surviving a discriminating-dose bioassay. The dark-blue line shows the posterior median of the estimated resistance trajectory and the light-blue shaded area shows its 95% credible interval. The wider grey shaded area represents the 95% posterior predictive interval, indicating the expected variation between individual bioassay observations. The upward trajectory was retained in each geographical analysis, although the estimated magnitude and uncertainty varied according to the observations included.

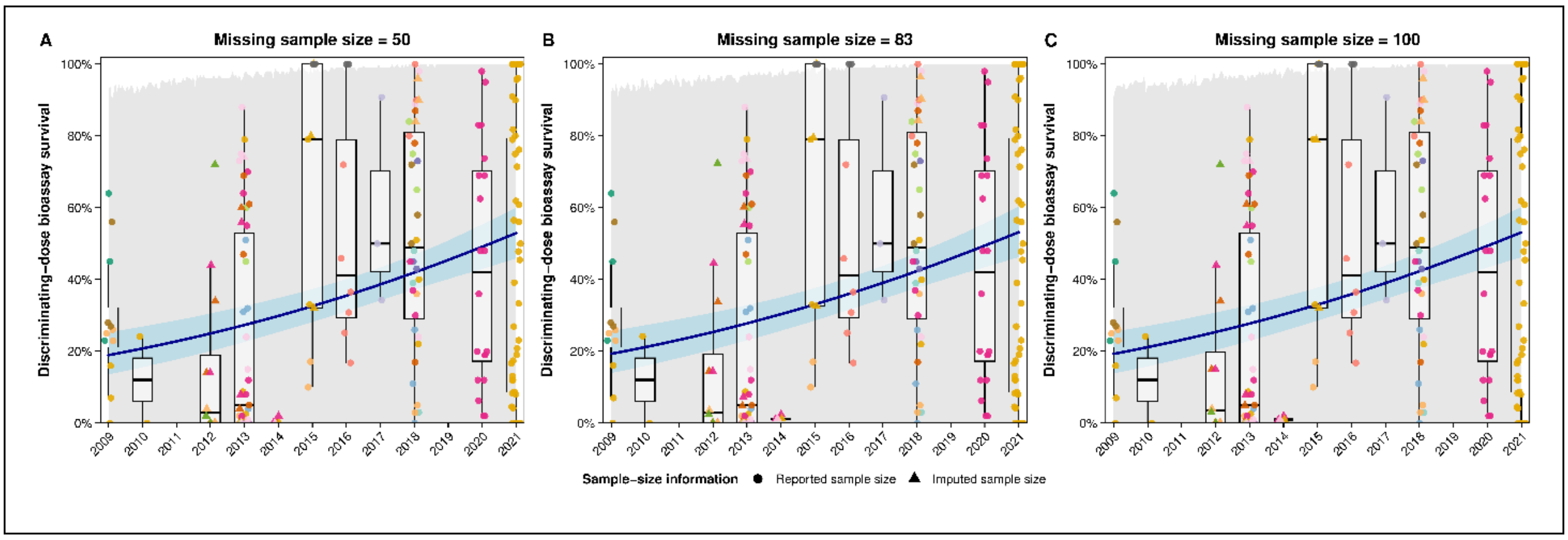

Supplementary Figure S2. Sensitivity of the estimated pyrethroid-resistance trajectory to the assumed sample size of bioassays with missing sample-size information. Missing sample sizes were imputed using three alternative values: 50 mosquitoes (**A**), 83 mosquitoes (**B**), and 100 mosquitoes (**C**). Points represent the observed proportion of mosquitoes surviving the discriminating-dose bioassay. Circles indicate bioassays for which the number of mosquitoes tested was reported, whereas triangles indicate bioassays for which the sample size was imputed. Boxplots summarise the distribution of observed bioassay survival in each year. The solid blue line represents the estimated national bioassay-survival trajectory, and the blue shaded region represents the associated 95% credible interval.

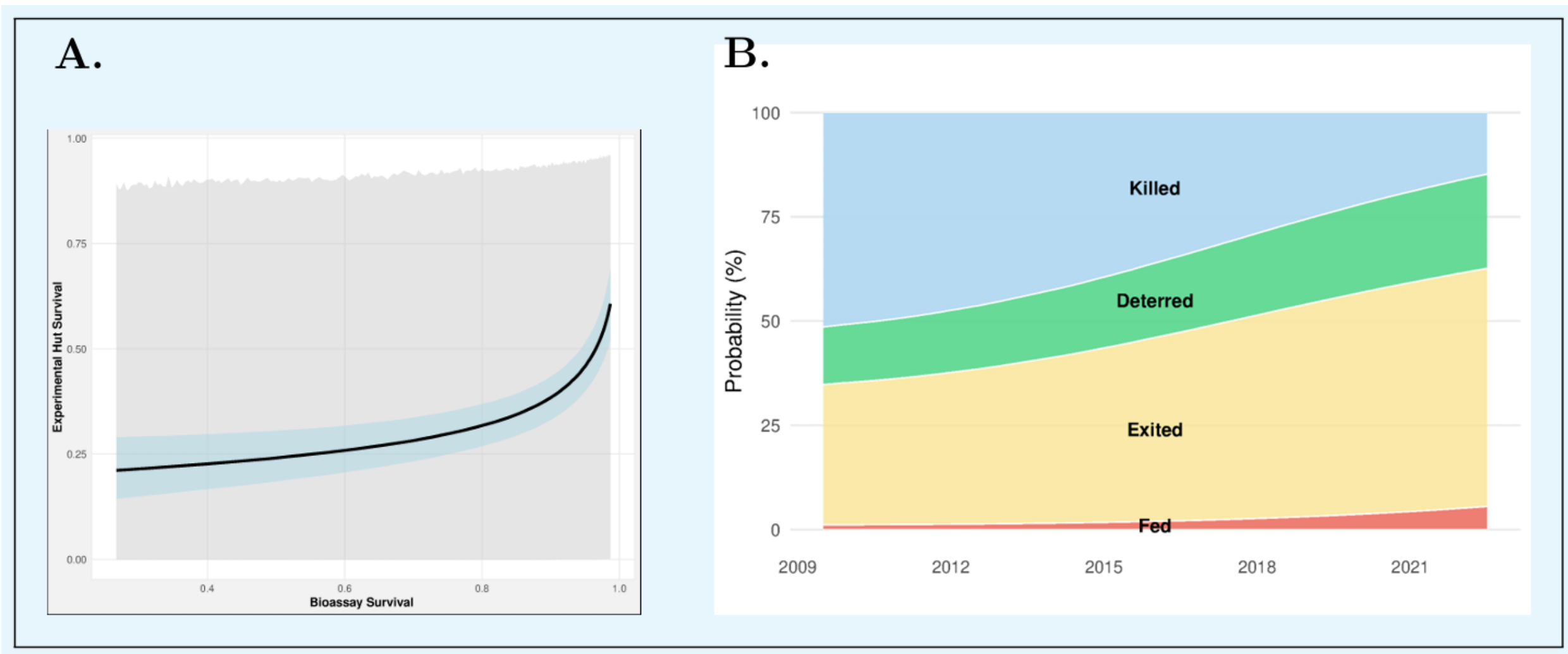

Supplementary Figure S3. **Bioassay-hut survival relationship and changes in mosquito outcomes with increasing resistance. (A)** The black curve represents the fitted relationship between mosquito survival in standard laboratory bioassays and survival observed in experimental hut trials. The light blue band shows the 95% credible interval from the posterior distribution, while the wider shaded band shows the 95% predictive interval from the beta-binomial model, capturing uncertainty in expected hut outcomes across resistance levels. **(B)** The stacked ribbons show how key mosquito outcomes in experimental huts change over time as resistance increases.

Supplementary Table S3. Parameters used to estimate the national pyrethroid-resistance trajectory and translate bioassay survival into experimental-hut outcomes.

Parameter	Description	Prior or source	Estimate (CrI)
**Parameters estimated in the national beta-binomial resistance model**			
a	Growth rate of the generalised logistic resistance trajectory	a ~ Lognormal(0.75, 0.75)	0.30 (0.13–0.67)
b	Inflection point of the generalised logistic resistance trajectory	b ~ Lognormal(1.0, 1.0)	15.57 (5.64–21.20)
c	Shape parameter controlling the asymmetry of the resistance trajectory	c ~ Lognormal(0.5, 0.5)	2.70 (0.57–6.89)
k	Beta-binomial precision parameter describing variation between bioassays	k ~ Pareto(1.0, 1.5)	1.23 (1.03–1.46)
**Parameters for deterrence model**			
δ_1	Scale parameter in the relationship between experimental-hut survival and deterrence	Published posterior distribution from Nash et al. (2021) and Churcher et al. (2024)	0.41 (0.32–0.53)†
δ_2	Shape parameter in the relationship between experimental-hut survival and deterrence	Published posterior distribution from Nash et al. (2021) and Churcher et al. (2024)	0.13 (0.00–0.66)†
δ_3	Curvature parameter in the relationship between experimental-hut survival and deterrence	Published posterior distribution from Nash et al. (2021) and Churcher et al. (2024)	1.49 (0.07–4.43)†
**Parameters for successful blood-feeding model**			
δ_4	Scale parameter in the relationship between experimental-hut survival and successful blood-feeding	Corresponds to θ_1 in Nash et al. (2021) and Churcher et al. (2024)	4.17 (3.88–4.49)†
δ_5	Curvature parameter in the relationship between experimental-hut survival and successful blood-feeding	Corresponds to θ_2 in Nash et al. (2021) and Churcher et al. (2024)	0.02 (0.01–0.03)†

C Supporting Results

C.1 Overall ITN use

Following the main analysis of overall ITN use shown in Figure 2A, the corresponding results for the remaining provinces are presented below.

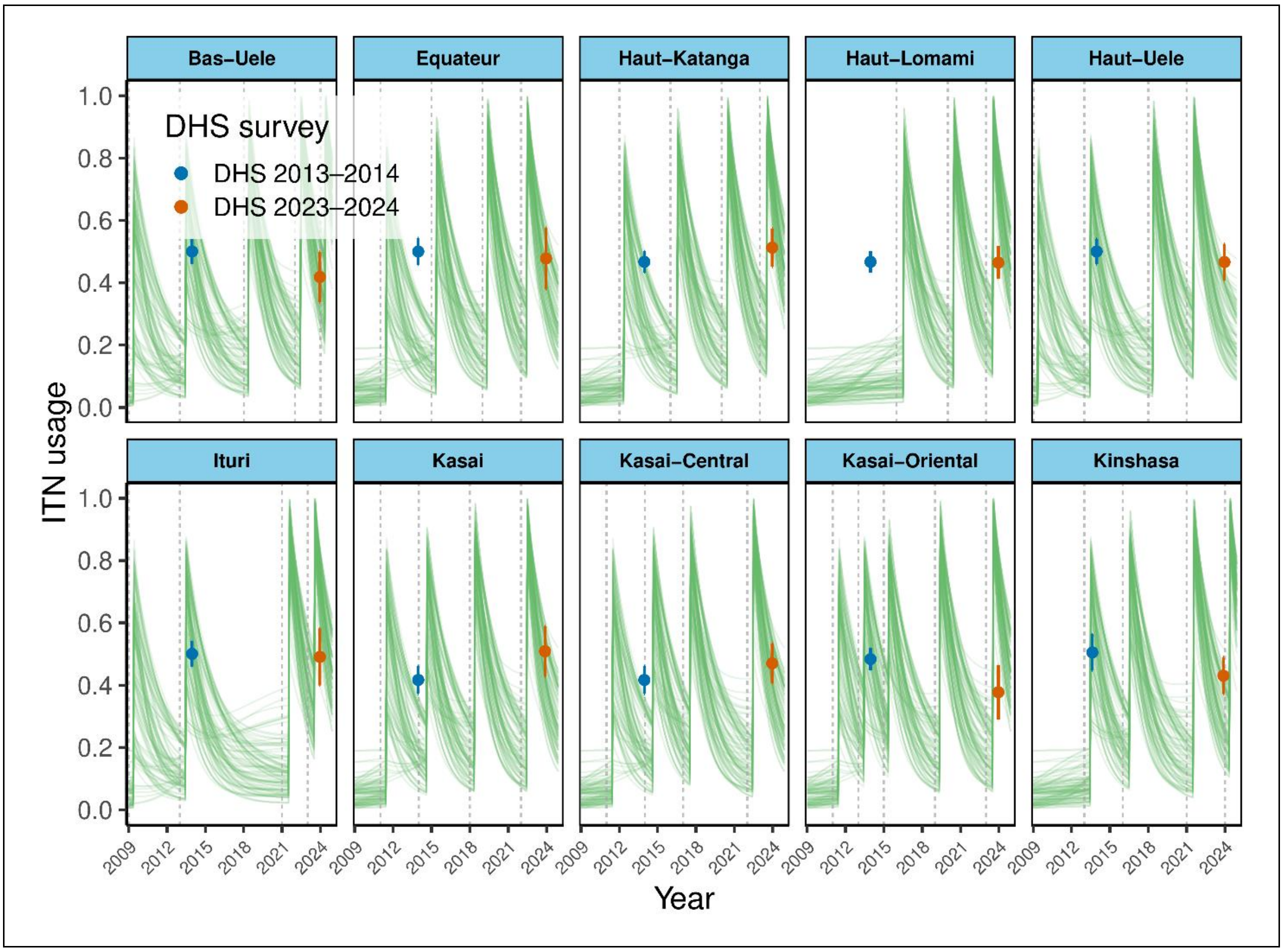

Supplementary Figure S4. Subnational time series of overall ITN use in the Democratic Republic of the Congo between 2009 and 2024.

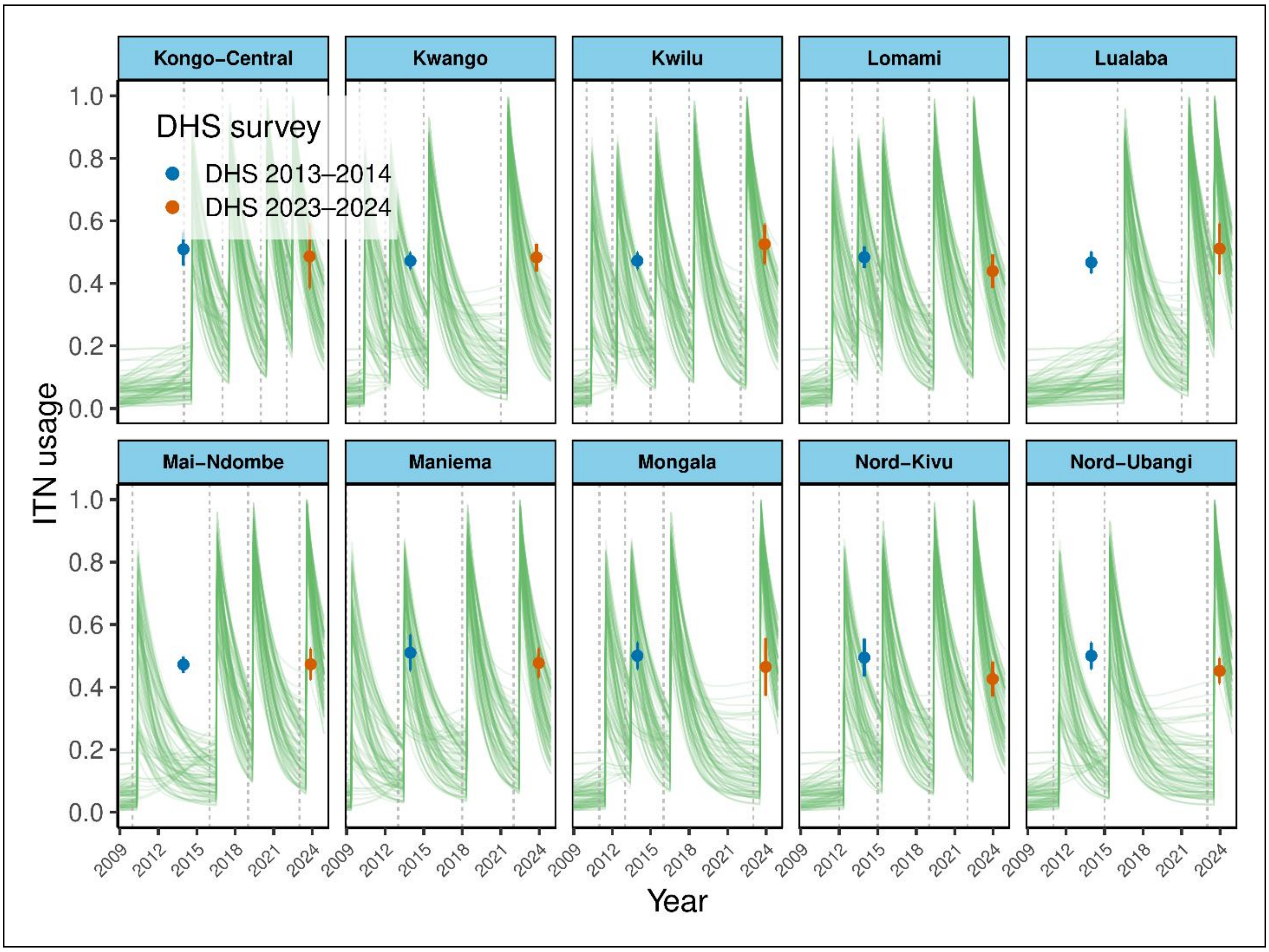

Supplementary Figure S5. Subnational time series of overall ITN use in the Democratic Republic of the Congo between 2009 and 2024.

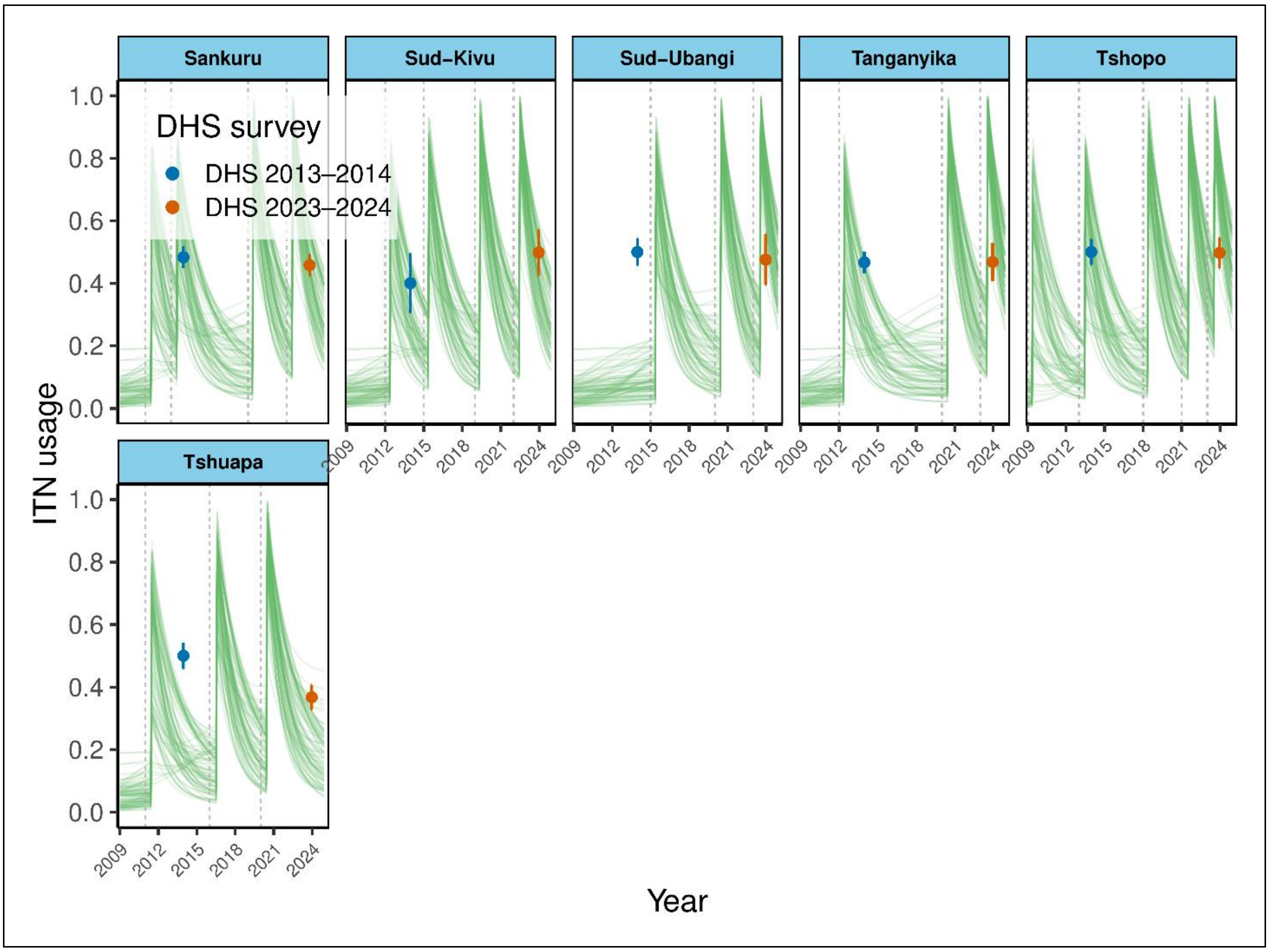

Supplementary Figure S6. Subnational time series of overall ITN use in the Democratic Republic of the Congo between 2009 and 2024.

Predicted malaria prevalence with or without resistance

After presenting the main analysis of predicted malaria prevalence with and without resistance in Figure 3A, we present below the corresponding results for the remaining provinces.

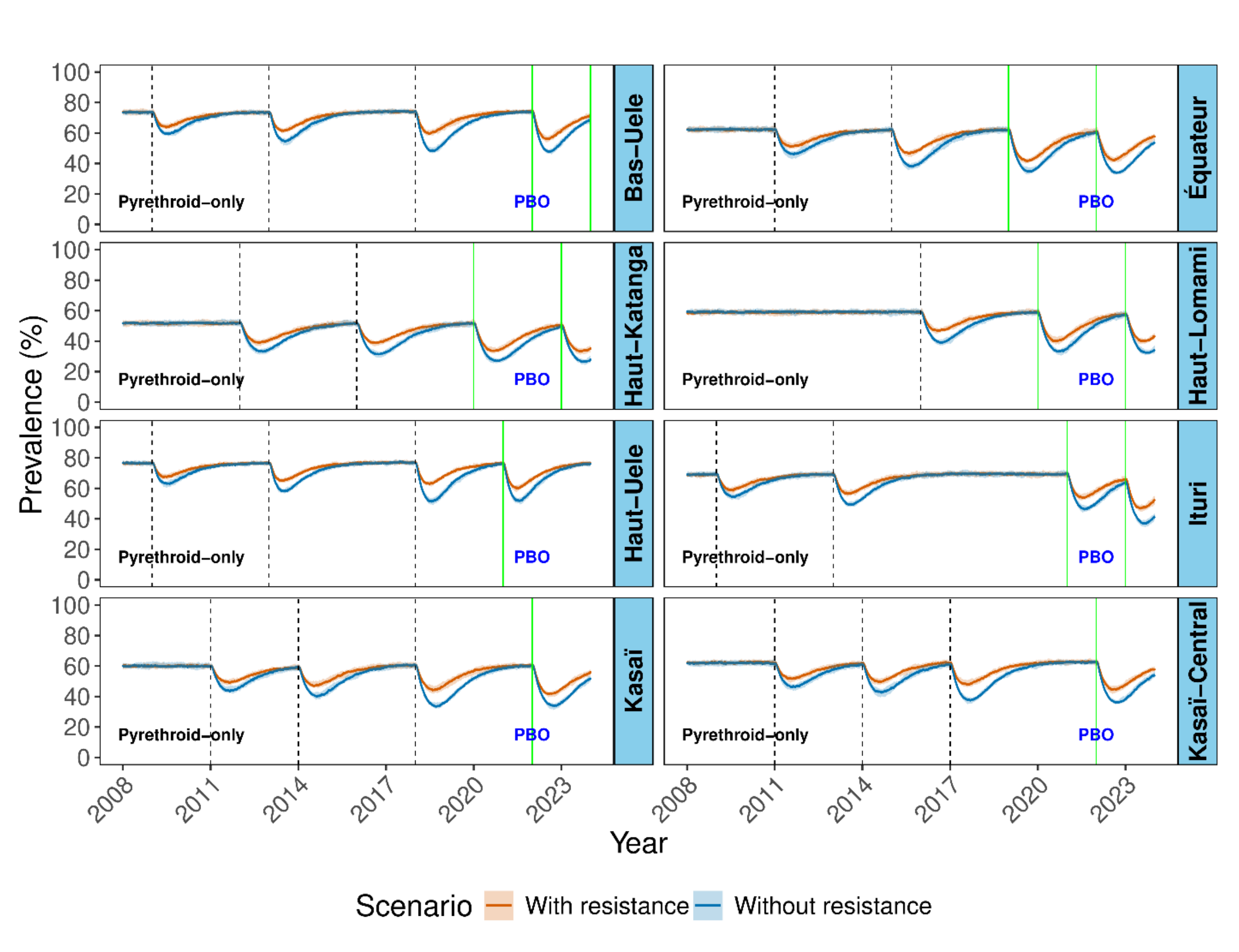

Supplementary Figure S7. Temporal trends in malaria prevalence under scenarios with and without pyrethroid resistance in the mosquito population. The orange line represents the reference scenario incorporating the estimated temporal change in pyrethroid resistance, while the blue line represents the counterfactual scenario in which mosquitoes remained fully susceptible to pyrethroids throughout the study period. Points show province-specific malaria prevalence estimates from the 2013–2014 and 2023–2024 Demographic and Health Surveys (DHS). Vertical dashed lines indicate mass distribution campaigns using pyrethroid-only ITNs, while solid green vertical lines indicate campaigns that introduced pyrethroid–PBO ITNs. Shaded bands represent the corresponding 95% credible intervals.

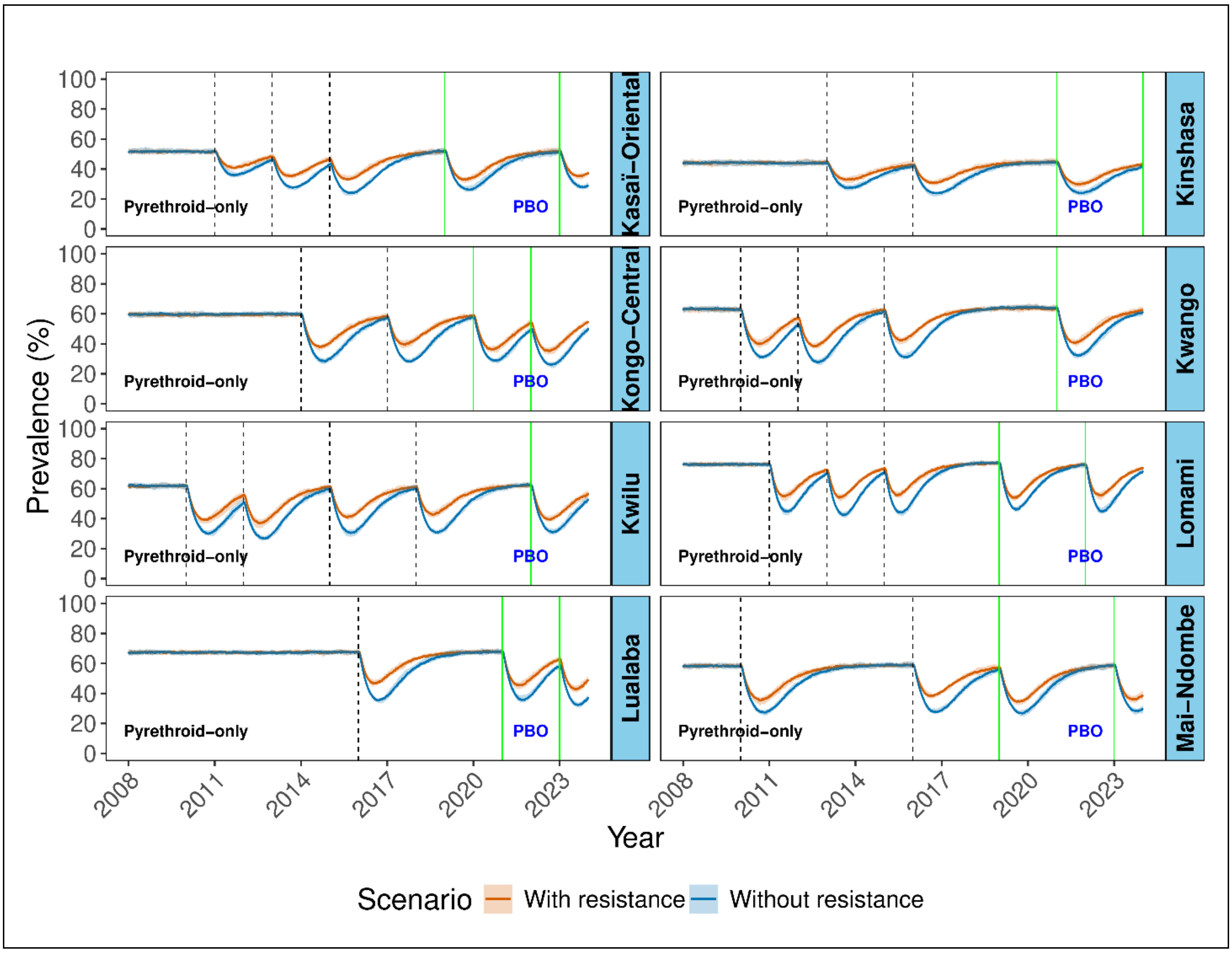

Supplementary Figure S8. Temporal trends in malaria prevalence under scenarios with and without pyrethroid resistance in the mosquito population. The orange line represents the reference scenario incorporating the estimated temporal change in pyrethroid resistance, while the blue line represents the counterfactual scenario in which mosquitoes remained fully susceptible to pyrethroids throughout the study period. Points show province-specific malaria prevalence estimates from the 2013–2014 and 2023–2024 Demographic and Health Surveys (DHS). Vertical dashed lines indicate mass distribution campaigns using pyrethroid-only ITNs, while solid green vertical lines indicate campaigns that introduced pyrethroid–PBO ITNs. Shaded bands represent the corresponding 95% credible intervals.

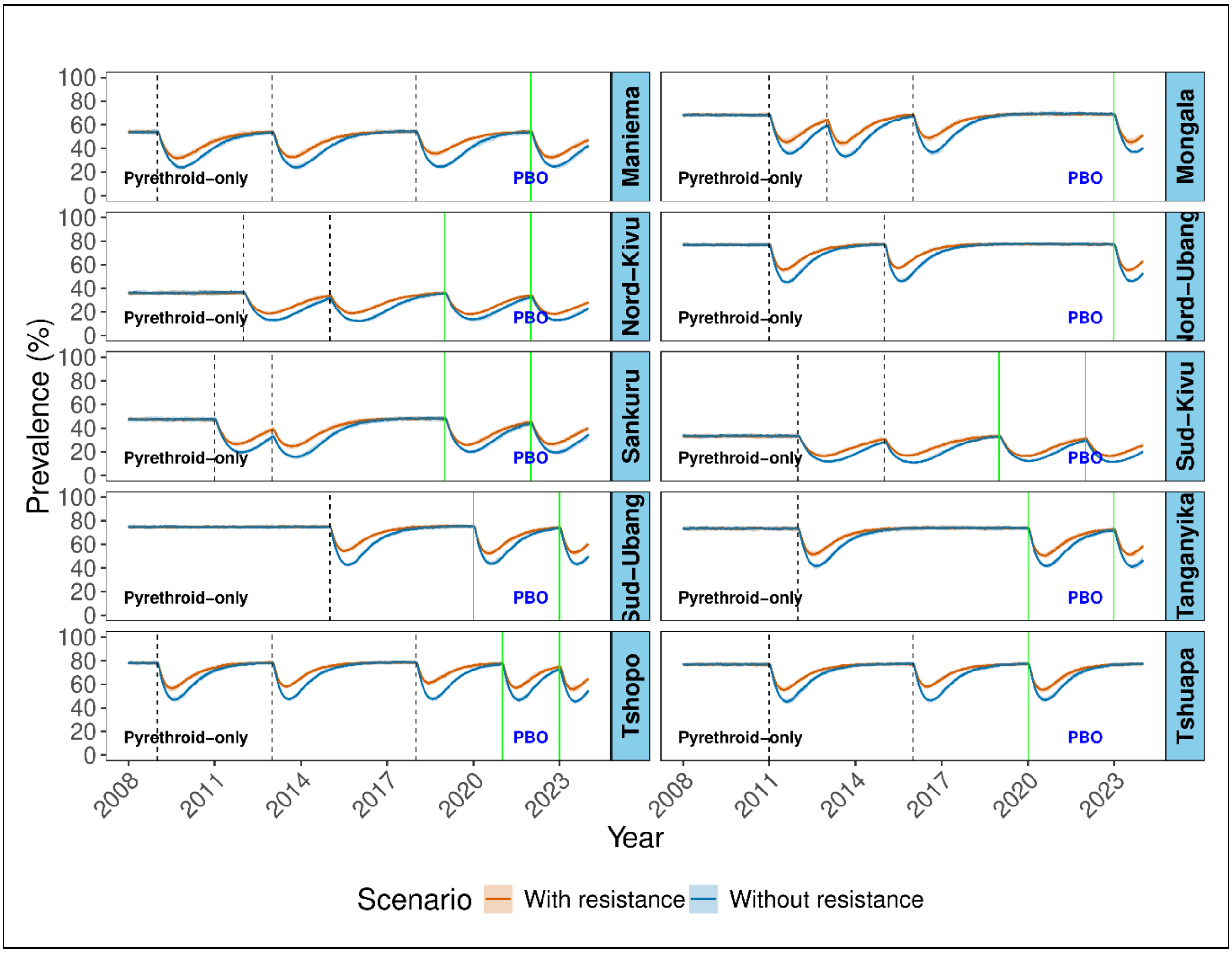

Supplementary Figure S9. Temporal trends in malaria prevalence under scenarios with and without pyrethroid resistance in the mosquito population. The orange line represents the reference scenario incorporating the estimated temporal change in pyrethroid resistance, while the blue line represents the counterfactual scenario in which mosquitoes remained fully susceptible to pyrethroids throughout the study period. Points show province-specific malaria prevalence estimates from the 2013–2014 and 2023–2024 Demographic and Health Surveys (DHS). Vertical dashed lines indicate mass distribution campaigns using pyrethroid-only ITNs, while solid green vertical lines indicate campaigns that introduced pyrethroid–PBO ITNs. Shaded bands represent the corresponding 95% credible intervals.

Cases averted due to ITN use across the 26 provinces

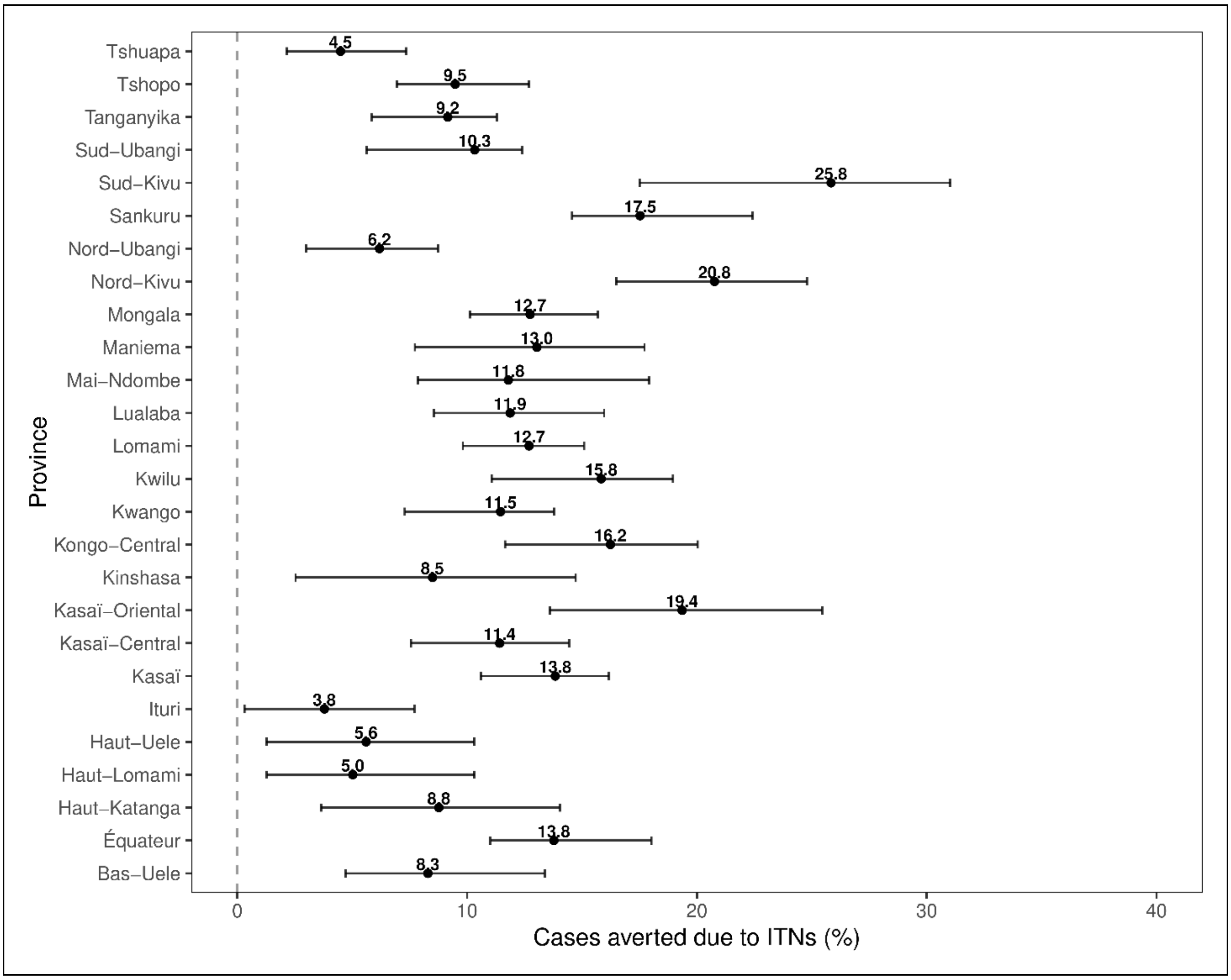

Supplementary Figure S10. Percentage of malaria cases averted by insecticide-treated net use across the 26 provinces of the Democratic Republic of the Congo.

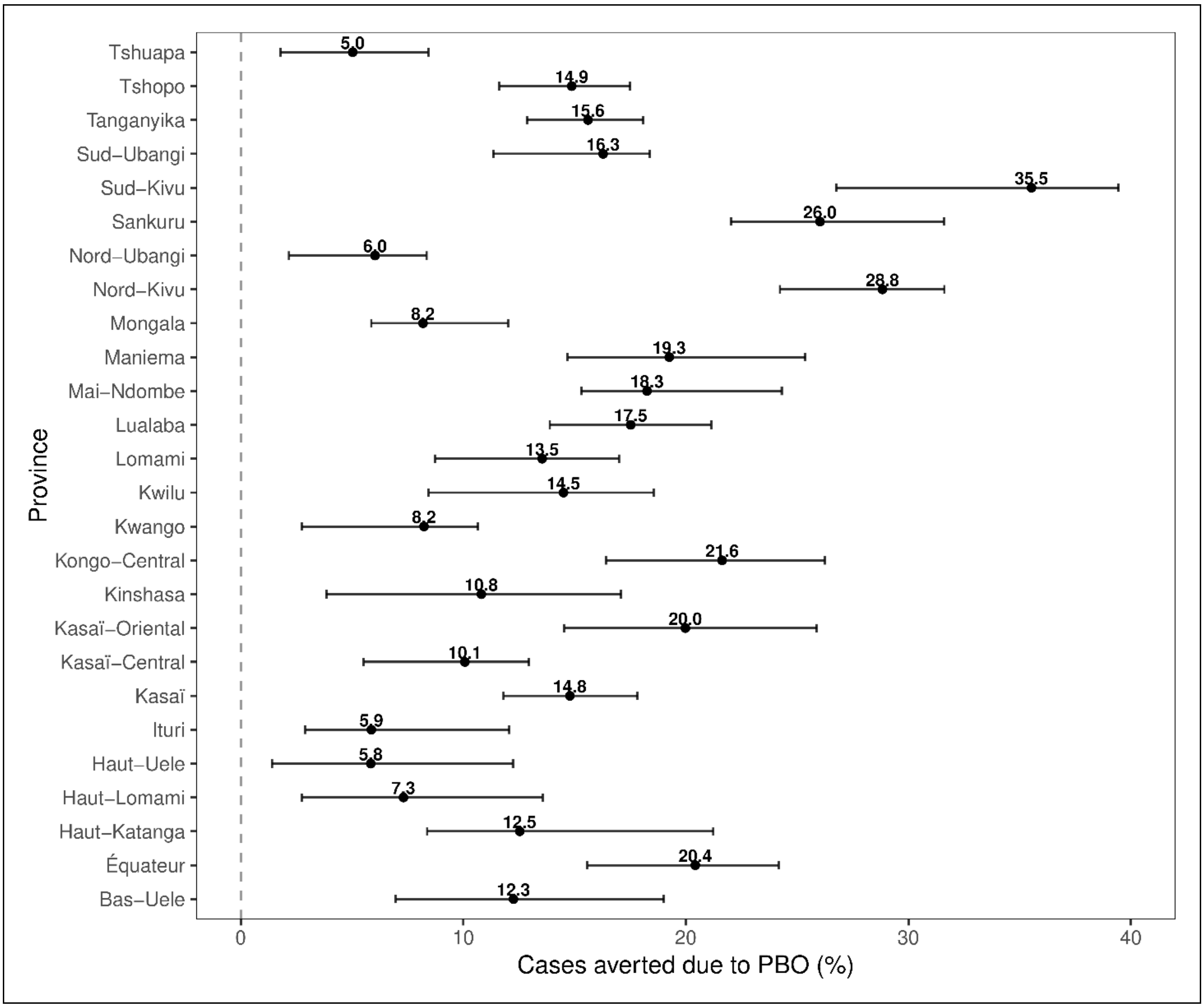

Supplementary Figure S11. Percentage of malaria cases averted due to pyrethroid–PBO insecticide-treated net use across the 26 provinces of the Democratic Republic of the Congo.

Deaths averted by ITN use

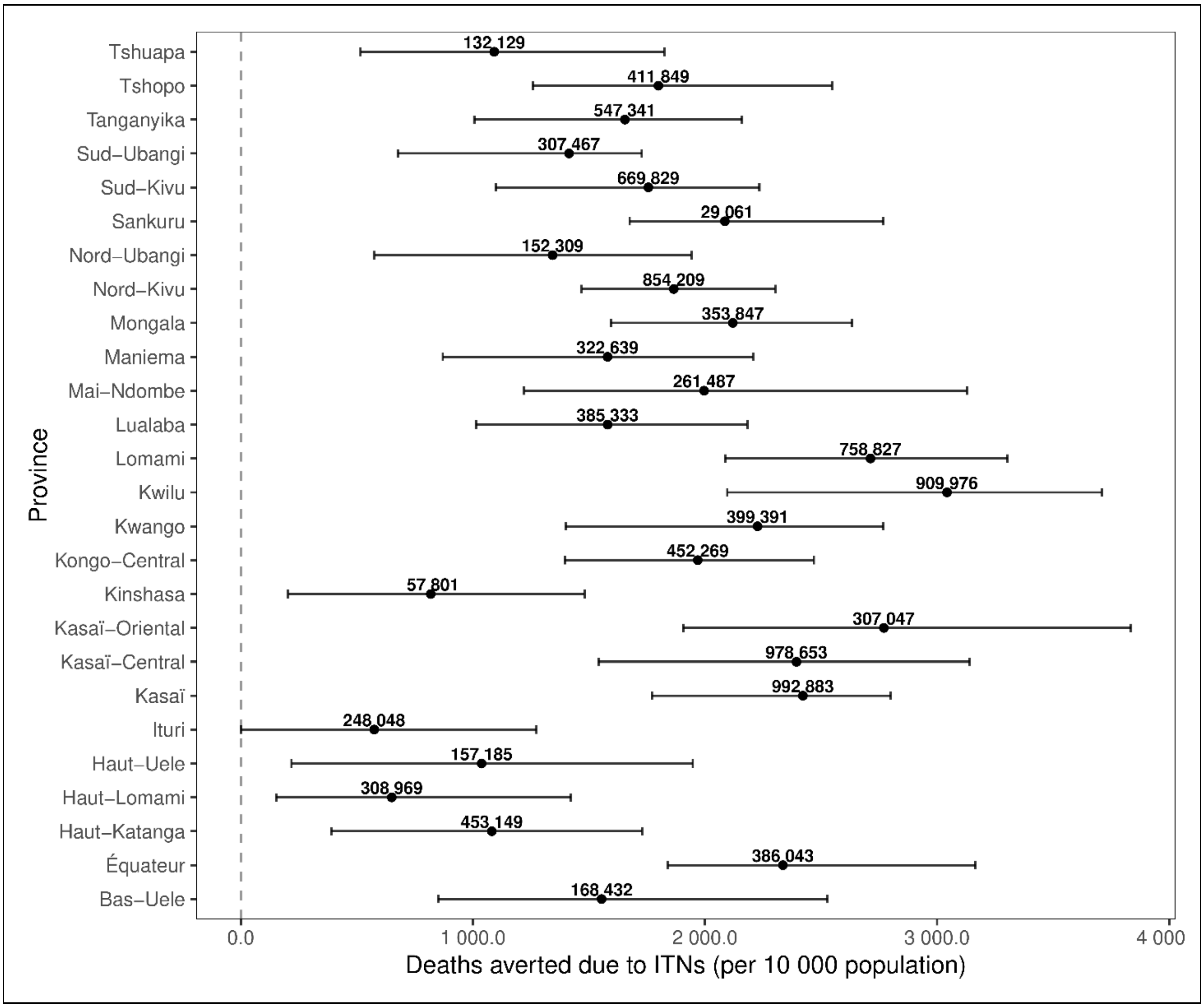

Supplementary Figure S12. Malaria deaths averted per 10,000 population by insecticide-treated net use across the 26 provinces of the Democratic Republic of the Congo. Numbers beside points indicate the corresponding cumulative number of deaths averted over 2009–2024.

Deaths averted by pyrethroid-PBO ITNs

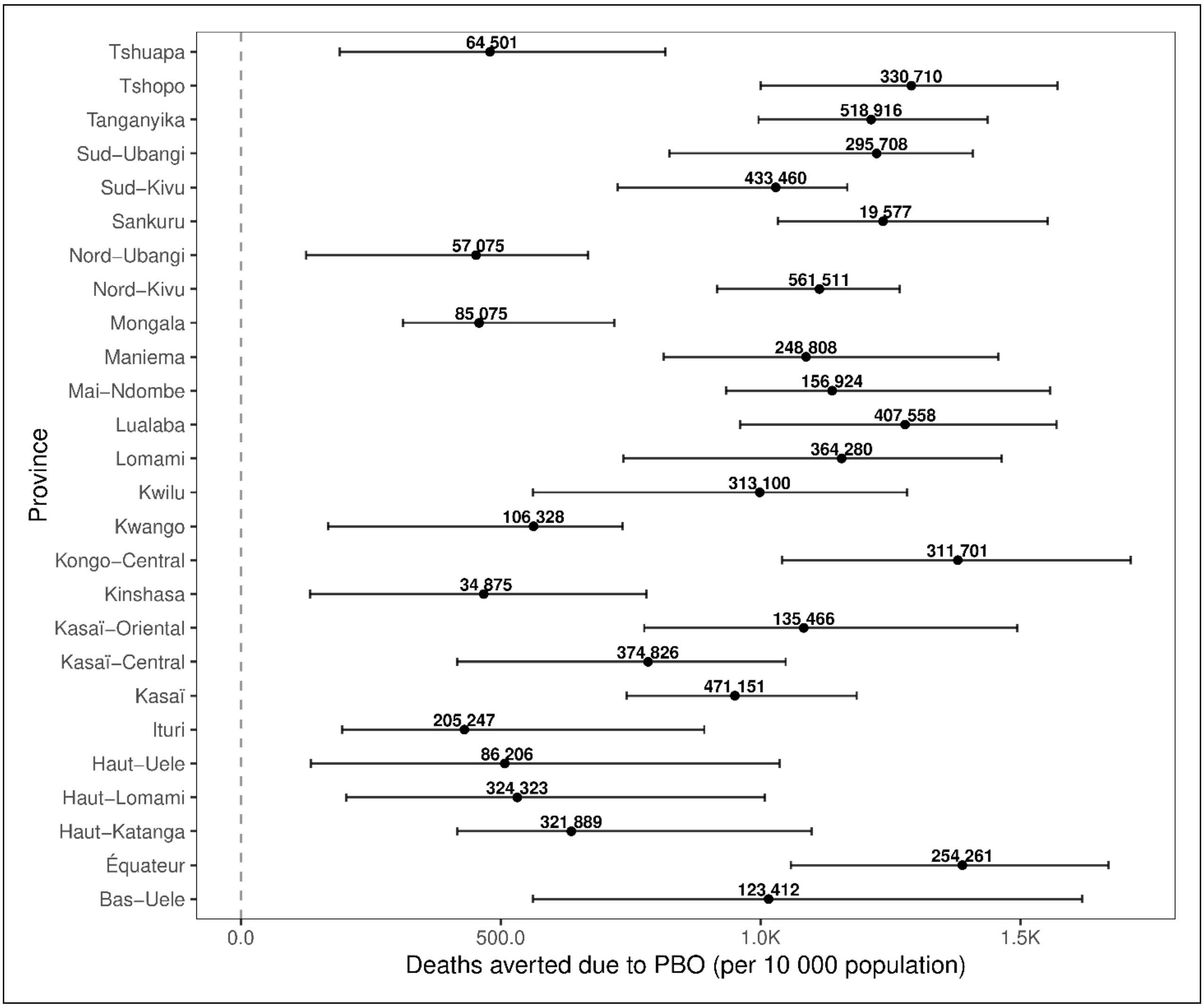

Supplementary Figure S13. Malaria deaths averted per 10,000 population by pyrethroid-PBO ITNs relative to the pyrethroid-only counterfactual across the 26 provinces of the Democratic Republic of the Congo. Numbers beside points indicate the corresponding cumulative number of deaths averted over 2019–2024.

Cases and deaths attributable to insecticide resistance

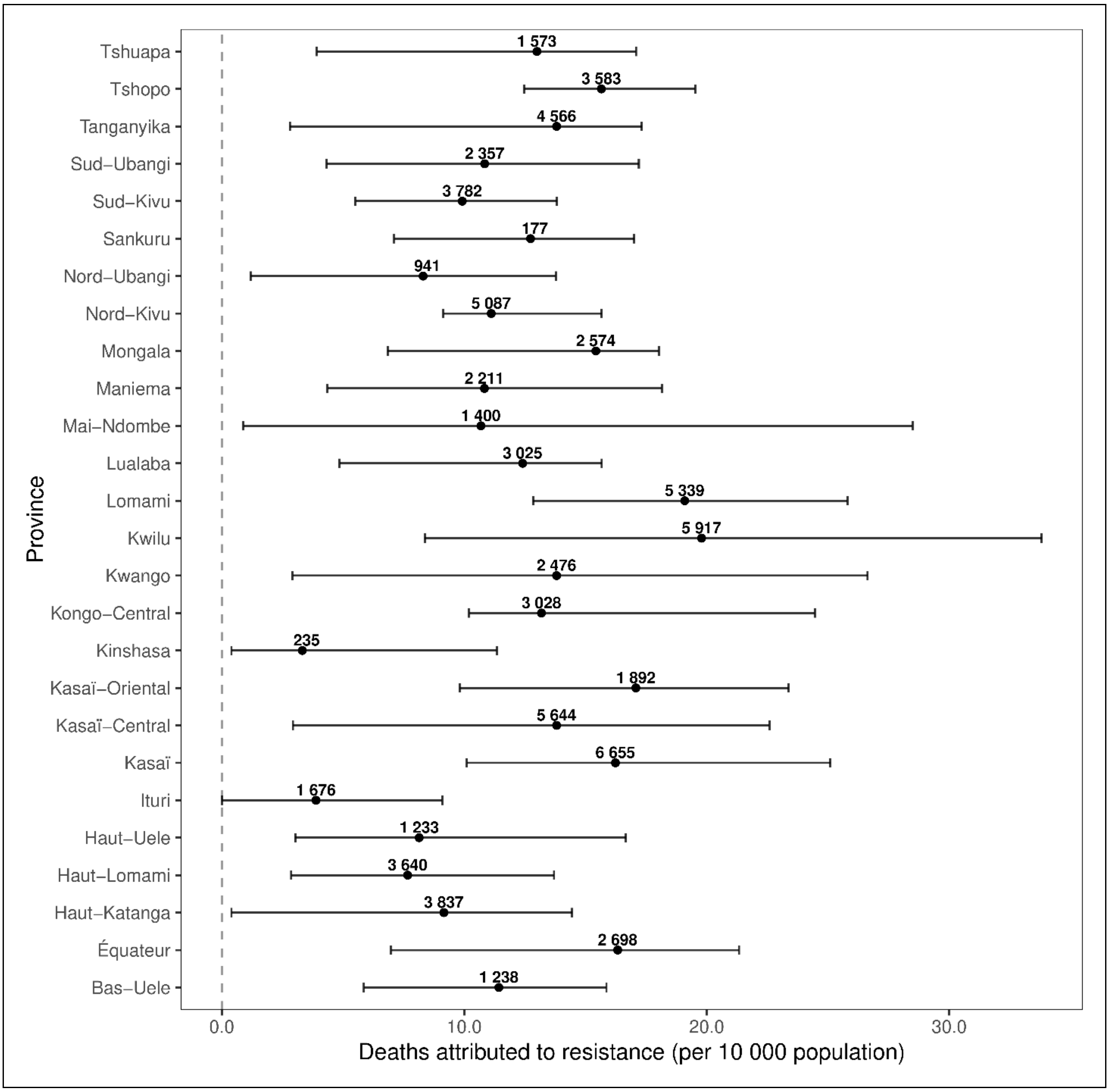

Supplementary Figure S14. Estimated malaria deaths attributable to pyrethroid resistance across the 26 provinces of the Democratic Republic of the Congo.

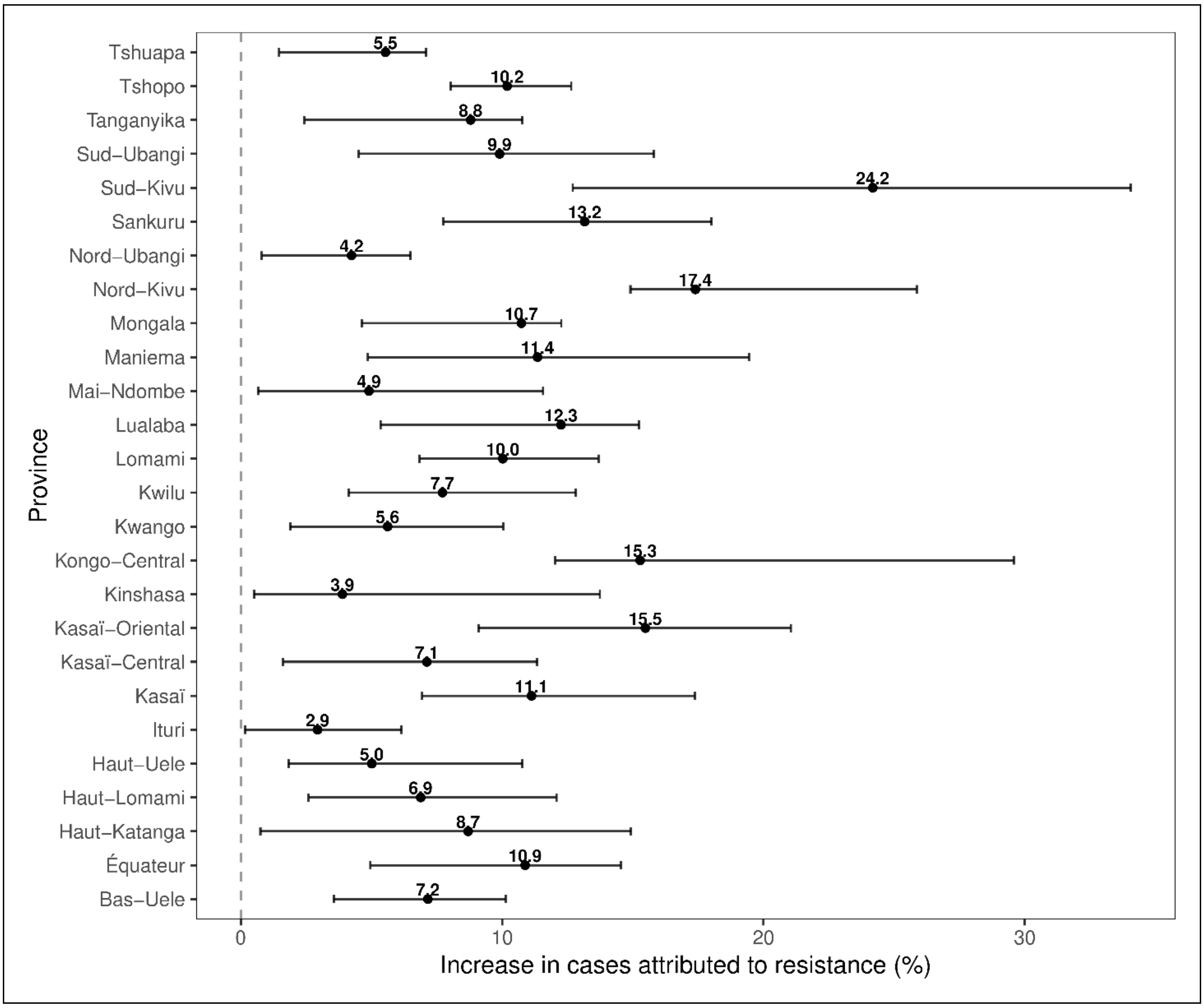

Supplementary Figure S15. Estimated increase in malaria cases attributable to pyrethroid resistance across the 26 provinces of the Democratic Republic of the Congo.

Provincial variation in ITN use in the DRC

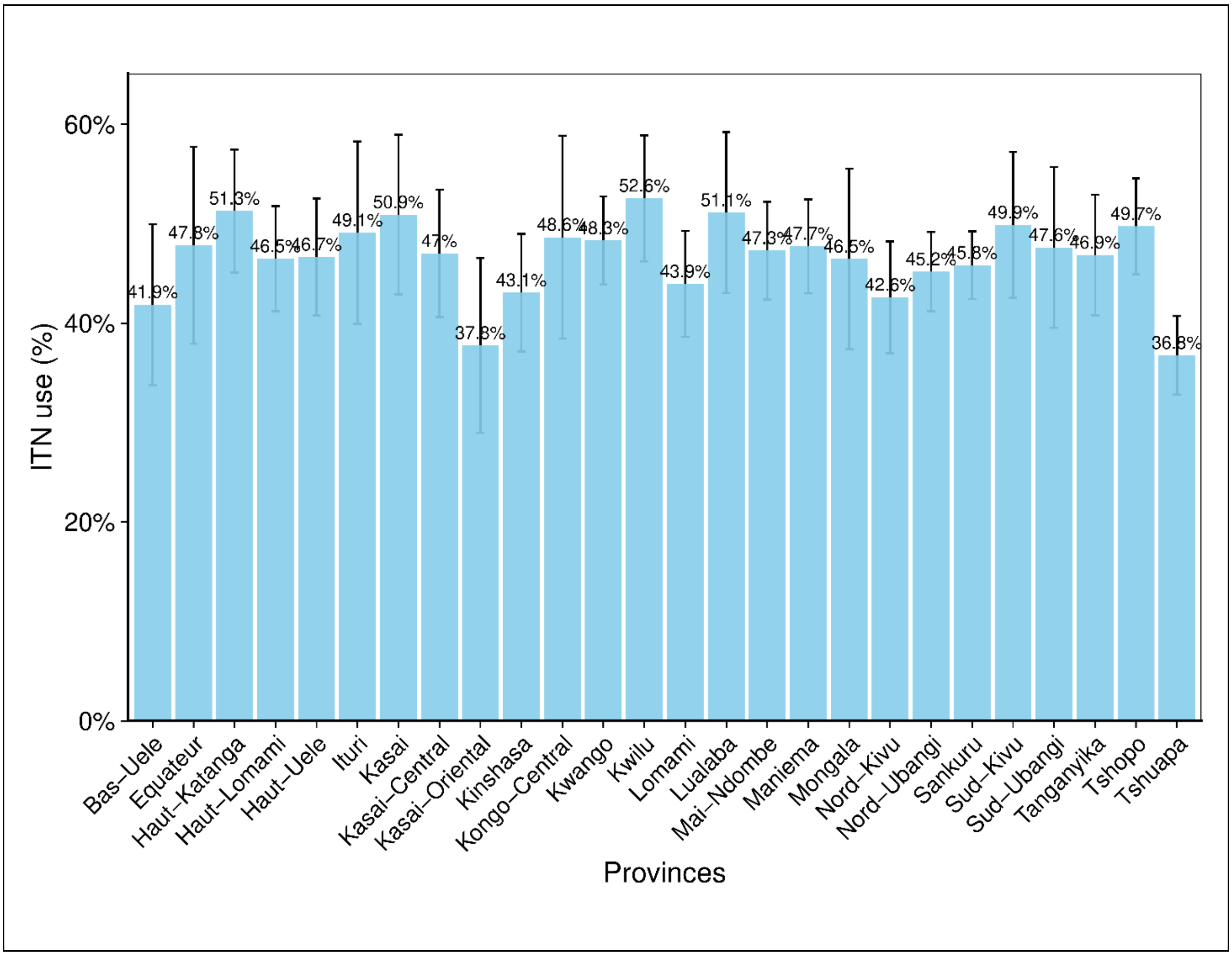

Supplementary Figure S16. Provincial estimates of ITN use in the Democratic Republic of the Congo.

ITNs distributed through routine channels in the DRC

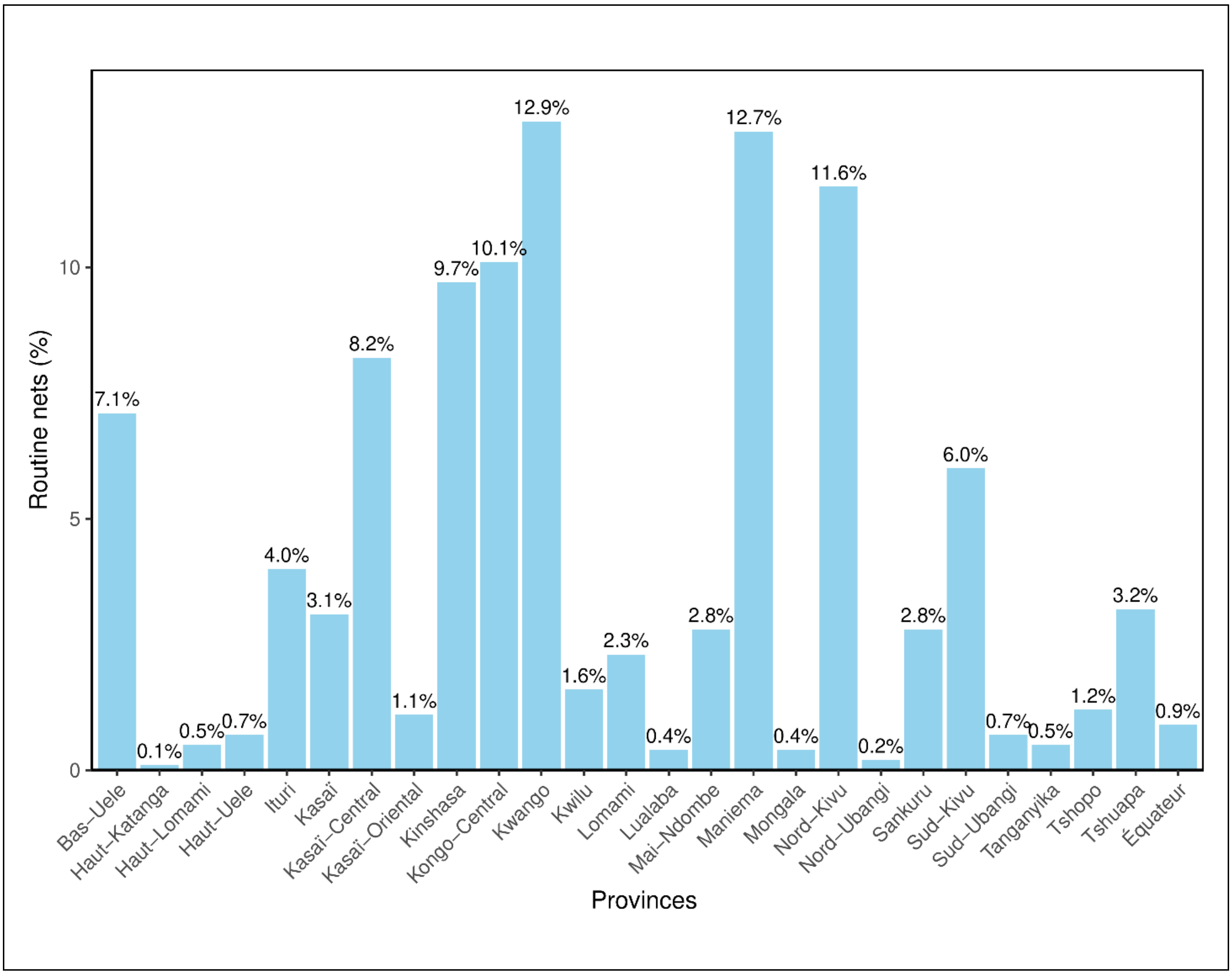

Supplementary Figure S17. Proportion of ITNs distributed through routine channels by province in the DRC.

Supplementary Table S4. National malaria cases and deaths after calibration to WHO estimates under ITN use with insecticide resistance in the Democratic Republic of the Congo, 2010–2024.

Year	Cases	Deaths	Case conversion factor	CFR
2010	26,144,344	85,123	0.0857	0.00326
2011	25,522,250	78,483	0.0216	0.00308
2012	24,598,662	64,511	0.0151	0.00262
2013	23,531,534	57,471	0.0104	0.00244
2014	23,314,218	60,156	0.00973	0.00258
2015	24,556,966	60,443	0.0101	0.00246
2016	26,049,693	66,971	0.00893	0.00257
2017	28,221,065	68,597	0.00902	0.00243
2018	30,384,274	77,570	0.00954	0.00255
2019	31,580,724	75,254	0.00970	0.00238
2020	32,532,899	75,025	0.00997	0.00231
2021	33,515,984	70,300	0.00976	0.00210
2022	33,580,334	70,024	0.0103	0.00209
2023	34,412,847	67,464	0.0111	0.00196
2024	35,175,261	67,676	0.00973	0.00192

DHS malaria RDT testing

Supplementary Table S5. Malaria rapid diagnostic test results by province in the 2013–2014 DHS, Democratic Republic of the Congo. The table reports the number of individuals tested and the number testing positive for malaria by RDT.

Province	Number tested	Number positive
Kinshasa	410	75
Bandundu	1,092	165
Bas-Congo	379	102
Équateur	1,324	285
Kasaï-Occidental	727	252
Kasaï-Oriental	842	247
Katanga	1,089	327
Maniema	491	184
Nord-Kivu	1,015	310
Orientale	1,105	329
Sud-Kivu	1,058	358

Supplementary Table S6. Malaria rapid diagnostic test results by province in the 2023–2024 DHS, Democratic Republic of the Congo. The table reports the number of individuals tested and the number testing positive for malaria by RDT across the 26 provinces.

Province	Number tested	Number positive
Kinshasa	3,593	865
Kongo Central	2,517	930
Kwango	3,229	1,156
Kwilu	1,668	876
Mai-Ndombe	600	395
Kasaï	1,504	852
Kasaï Central	1,792	1,011
Kasaï Oriental	1,660	905
Lomami	1,438	848
Sankuru	1,342	763
Maniema	1,511	851
Sud-Kivu	2,302	1,079
Nord-Kivu	2,217	1,035
Ituri	1,789	962
Haut-Uele	1,209	689
Tshopo	2,041	1,085
Bas-Uele	1,031	586
Équateur	1,418	824
Mongala	1,277	768
Nord-Ubangi	1,090	657
Sud-Ubangi	1,164	694
Tshuapa	1,033	602
Haut-Katanga	1,836	690
Haut-Lomami	1,292	741
Lualaba	1,147	509
Tanganyika	1,060	572
